# Combinational studies of BOLD-100/KP1339 with established chemotherapeutics in gastrointestinal multicellular tumor spheroids

**DOI:** 10.1007/s00280-026-04902-z

**Published:** 2026-05-22

**Authors:** Dominik Wenisch, Slavica Ždravac, Michael A. Jakupec, Franz Jirsa, Bernhard K. Keppler

**Affiliations:** 1 Institute of Inorganic Chemistry, Faculty of Chemistry, University of Vienna, Währinger Strasse 42, 1090 Vienna, Austria; 2 Research Cluster “Translational Cancer Therapy Research”, University of Vienna, Währinger Strasse 42, 1090 Vienna, Austria; 3 Institute of Inorganic Chemistry, Faculty of Chemistry, University of Vienna, Josef-Holaubek-Platz 2, 1090 Vienna, Austria

**Keywords:** Colon cancer, Combination therapy, Gastric cancer, Multicellular tumor spheroids, Ruthenium drug, In vitro studies

## Abstract

BOLD-100, a ruthenium(III) prodrug also known as KP1339, is currently under clinical investigation for its suitability as a co-therapeutic anticancer drug. Unlike platinum-based chemotherapeutics, it has unique non-DNA targets, mainly GRP78, an enzyme responsible for signaling related to protein folding in the ER. Inhibition of this key mediator of the “unfolded protein response” may lead to cell death eventually. The purpose of this study was to assess in vitro the impact of BOLD-100 combinations with oxaliplatin, 5-FU, cisplatin or SN38 in multicellular tumor spheroids (MCTSs) compared to single-drug treatments. Gastric (MKN45, NCI-N87) and colorectal cancer (HCT116, HT29) cell lines were chosen, corresponding to the clinical trial in which patients with tumors of these origins are being treated. Biological effects investigated in this study include cytotoxic activity, synergism/antagonism based on Chou and Talalay’s algorithm, formation of reactive oxygen species (ROS) and induction of apoptosis and necrosis. Cytotoxicity tests showed vastly different chemosensitivities in MCTSs of the same tumor origin. In both tumor entities, partially synergistic effects were revealed when BOLD-100 was combined with the drugs mentioned above. The DCFH-DA assay suggested consistent increases of ROS levels after treatment with oxaliplatin and, with restrictions, its combination with BOLD-100. Apoptosis and necrosis were induced in the spheroid models by single-drug and combined treatment, with no hints at antagonism in the combination settings. In conclusion, these findings emphasize the potential of BOLD-100 for combination therapy of gastric and colorectal cancers.

**Supplementary Information:**

The online version contains supplementary material available at 10.1007/s00280-026-04902-z.

## Introduction

Chemotherapy is a powerful treatment modality for therapy of various solid tumor entities. Platinum-based drugs, such as cisplatin, oxaliplatin and carboplatin, are still among the most frequently applied cancer chemotherapeutics [1]. Beside their property of attacking cancer cells, adverse effects, such as neurotoxicity, nephrotoxicity or myelosuppression [2], occur frequently due to unspecific interactions with normal cells, thereby limiting patients’ tolerance to these therapeutic approaches.

A well-established alternative to single drugs is their combined application. Enhancement of therapeutic impact of a potent drug with simultaneous diminution of adverse effects by one or more auxiliary drug(s) might be an optimal combinational strategy [3–5]. Beside platinum-based chemotherapeutics, various ruthenium (Ru) complexes have shown anticancer activity in vitro and in vivo, and a few even have proceeded to the stage of clinical evaluation [6–8]. BOLD-100, a Ru(III) pro-drug also known as KP1339, is currently investigated in patients with advanced gastrointestinal tumors in a clinical phase II trial (NCT04421820) [9]. Over the course of the trial, this Ru-based drug already showed promising beneficial effects in combination with the FOLFOX regimen, pre-dominantly in colorectal cancer patients as well as in patients with gastric or biliary tract cancer [10–12]. Frequently used for various cancer therapies, FOLFOX is a triple compound combination, consisting of folinic acid, 5-fluorouracil and oxaliplatin, with 5-fluorouracil and oxaliplatin being the cytostatic components and folinic acid a biological response modifier enhancing 5-fluorouracil’s activity. Both cytostatic drugs exert their activity mainly through interference with DNA and RNA functionality in the nucleus. Whereas 5-fluorouracil acts as an antimetabolite hindering transcription and replication by its insertion instead of the nucleobases uracil (RNA) and thymidine (DNA) into nucleic acids, oxaliplatin (like cisplatin) inhibits DNA transcription by cross-linking nucleobases [13, 14]. BOLD-100, on the other hand, affects endoplasmic reticulum (ER) function as well as ribosome biogenesis and interferes with glycolysis. Its inhibitory effect on the ER chaperone GRP78 counteracts the “unfolded protein response” (UPR), causing ER stress by accumulation of not properly folded proteins; additionally, BOLD-100 strongly interferes with glycolytic activity, likewise leading to cell death [15–18]. Combining these differently acting drugs may be an effective strategy for attacking cancer cells, but also for mitigating adverse effects for cancer patients. Based on its unique mode of action and a toxicity profile hardly overlapping with those of other cytostatic drugs, BOLD-100 had already been identified as a prime candidate drug for combination therapies [19]. Indeed, patients with metastatic colorectal cancer, who had been pre-treated heavily, have shown remarkable tolerance with minimal neuropathy or other toxicities after combined treatment with BOLD-100 plus FOLFOX [10].

Under these aspects, we designed our study to investigate fundamental cellular responses in multicellular tumor spheroids (MCTSs) [20, 21] of gastrointestinal cancer cell lines to deepen the knowledge on dual-drug combinational effects of BOLD-100 with oxaliplatin, 5-FU, cisplatin or SN38. The cell lines MKN45, N87, HCT116 and HT29 were chosen from a variety of cell lines representing carcinomas of gastric or colorectal origin. First, we investigated the principal generation and growth of MCTSs from these four cell lines to confirm their suitability for this study. Upon establishment of spheroids, cellular impacts of BOLD-100 in combination with either 5-FU or oxaliplatin (both components of the FOLFOX regimen) were investigated in all four cell lines. Additionally, the combination of BOLD-100 with cisplatin was tested in spheroids of the gastric cancer cell lines MKN45 and N87, and the combination of BOLD-100 with SN-38 (the metabolically active form of irinotecan, a topoisomerase I inhibitor) in those of colorectal cell lines HCT116 and HT29, based on the frequent application of cisplatin and irinotecan for therapy of these tumor entities, respectively [22–24].

Pivotal cellular effects investigated in this study include cytotoxicity and its synergism/antagonism in combination settings, the generation of reactive oxygen species (ROS) (primarily due to BOLD-100’s redox-active metal center), and the induction of apoptosis and necrosis.

## Materials and methods

### Human carcinoma cell culture and spheroid formation

Gastric carcinoma cell lines NCI-N87 (in the following abbreviated as “N87”) and MKN45 were purchased from the American Type Culture Collection (ATCC) and the German Collection of Microorganisms and Cell Cultures (DSMZ), respectively. Both cell lines were maintained in RPMI 1640 medium (*Sigma Aldrich*, St. Louis, USA), supplemented with 10% heat inactivated fetal calf serum of Mexican origin (*Serana*, Pessin, Germany) and 4 mM L-glutamine (*Sigma Aldrich*, St. Louis, USA).

Colorectal carcinoma cell lines HCT116 and HT29 were kindly provided by the Center for Cancer Research, Department of Medicine I, Medical University of Vienna, Austria. HCT116 and HT29 cells were maintained in McCoy’s 5a medium (*Sigma Aldrich*, St. Louis, USA), supplemented with 10% fetal calf serum and 4 mM L-glutamine.

Cell lines used in this work have been authenticated *via* STR profiling in June 2022 (*Microsynth*, Balgach, Switzerland).

All cells were cultured as adherent monolayers in 75 cm^2^ flasks (*CytoOne*, *Starlab*, Hamburg, Germany) and kept in a humidified incubator at 37 °C with 5% CO_2_.

For multicellular tumor spheroid formation, cell lines were harvested after trypsinization and seeded in 96-well ultra-low-attachment (ULA) plates (*Corning*, New York, USA). Each well containing cells was allowed to form a single spheroid at 37 °C in a 5% CO_2_ atmosphere within 96 h.

### Compounds

Sodium *trans*-[Tetrachloridobis(1 *H*-indazole)ruthenate(III)] (BOLD-100) was synthesized at the Institute of Inorganic Chemistry, University of Vienna, and maintained under Ar atmosphere until usage. A stock solution in 100% DMSO was prepared, which was immediately diluted in the respective culture medium, with the organic solvent concentration not exceeding 0.5% (v/v) when treatment of the spheroids was performed.

[(1*R*,2*R*)-Cyclohexane-1,2-diamine](ethanedioato-*O*,*O*’)platinum(II) (oxaliplatin) was purchased from BLD Pharmatech Ltd. (Cat. Nr.: BD146411). A 100% DMSO stock was prepared, which was immediately diluted in the respective culture medium, with the organic solvent concentration not exceeding 0.5% (v/v) when treatment of the spheroids was performed.

5-Fluor-1,2,3,4-tetrahydropyrimidin-2,4-dion (5-fluorouracil) was purchased from Thermo Fisher (Cat. Nr.: 228440010). A stock solution in sterilized de-ionized H_2_O was prepared, which was diluted in respective culture medium subsequently at least ten times when treatment of the spheroids was performed.

*cis*-Diamminedichloridoplatinum(II) (cisplatin) was synthesized at the Institute of Inorganic Chemistry, University of Vienna. Cisplatin was dissolved and diluted in supplemented RPMI 1640 medium when treatment of the spheroids was performed.

7-Ethyl-10-hydroxycamptothecin (SN38) was purchased from Thermo Fisher (Cat. Nr.: 459091000). A stock solution in 100% DMSO was prepared, which was diluted in the respective culture medium subsequently, with the organic solvent concentration not exceeding 0.5% (v/v) when treatment of the spheroids was performed. Concentrations of > 20 µM SN38 in DMSO/medium should be taken with caution, due to solubility issues.

### Formation of multicellular tumor spheroids

For all cell lines, different cell numbers were seeded to obtain compact spheroids with sizes between 250 and 500 μm. In this range, spheroids are supposed to be compact enough, while containing viable cells pre-dominantly [25]. Tested cell lines were seeded in cell numbers of 0.25 × 10^3^, 0.5 × 10^3^, 2.5 × 10^3^ and 5.0 × 10^3^ cells per well in 100 µL medium, and every day pictures were taken for a total period of 8 days with an Olympus (*CKX41)* inverse microscope with 4× objective magnification, phase contrast and a ColorView camera. Two orthogonal diameters of three spheroids per condition were measured with the *Cell-F* software for statistical evaluation. *Friedrich et al.* recommended a period of 96 h for pre-forming equally-sized spheroids before the start of drug screening experiments [26]. After images were taken on day 4, further 100 µl per well of the respective medium for each cell line were added to maintain cell viability and growth properties. From this point onward, all cell lines have been tested under same conditions regarding growth time, renewal of medium and duration of treatment for reasons of comparability.

### Characterization of spheroids by confocal microscopy

For all subsequent experiments, proliferation and viability of cells within the spheroids were pivotal factors for testing the impact of chemotherapeutic drugs. Hence, immunofluorescence was applied for further characterization of untreated spheroids *via* confocal microscopy. 2.5 × 10^3^ cells in 100 µL per well were seeded into ULA plates for N87 cells, while 0.5 × 10^3^ cells per well were seeded for both colorectal cell lines (HCT116 and HT29). Spheroids grew for 4 days (96 h) and 8 days (192 h), with medium renewal after 96 h for the latter spheroids. After those incubation times, at least six spheroids were collected and transferred to biopsy cryomolds (10 mm × 10 mm × 5 mm, *Sakura Finetek*), embedded in TissueTek™ (*Sakura Finetek*) and frozen at − 20 °C. Afterwards, cryo-samples were cut into 5 μm thin sections with a cryomicrotome (CM3050 S, *Leica*) and mounted on superfrost glass slides (*Thermo Fisher Scientific*).

For immunostaining of those sections, samples were fixed with 4% paraformaldehyde (in PBS) for 30 min at room temperature and washed 3× with PBS. Afterwards, spheroids were permeabilized with 0.1% TritonX-100 (in PBS) for 10 min and washed 3× with PBS again. Then, spheroid samples were encircled with a grease pen for reducing consumption of blocking and antibody solutions. Samples were blocked with 10% goat serum + 2% bovine serum albumin (BSA, taken from a 5% stock solution/PBS) in PBS for 1 h, washed 3× with PBS and primary antibodies were added overnight at 4 °C in the dark. Samples were either incubated with KI-67 Rabbit mAb (diluted 1:600 in PBS/2% BSA, #9129, *Cell Signaling Technology*) for proliferating cells or HIF-1α Rabbit mAb (diluted 1:1000 in PBS/2% BSA, #36169, *Cell Signaling Technology*) for detection of hypoxic areas within the spheroids.

On the next day, samples were washed 3× with PBS and incubated for 1–1.25 h at room temperature with the secondary antibody (diluted 1:1000 in PBS/2% BSA, #8889, Anti-Rabbit IgG Fab 2 Alexa Fluor 594, *Cell Signaling Technology*). For the last step, samples were washed 3× with PBS once more and mounted with ProLong^TM^ Antifade Gold containing DAPI (P36941, *Invitrogen*) on cover slips. Prepared samples were stored at −80 °C until images were taken with a confocal microscope (LSM800, *Zeiss*).

### Cytotoxicity of single drugs as well as combined drugs in spheroid cell culture

Cytotoxicity was measured by means of the resazurin assay upon 96 h of treatment. For MKN45 and N87 spheroids, 0.25 × 10^3^ and 2.5 × 10^3^ cells in 100 µL per well were seeded into ULA plates, respectively. For the colorectal cell lines HCT116 and HT29, 0.5 × 10^3^ cells in 100 µL per well were seeded into ULA plates. Spheroids were allowed to form within the first 96 h, with treatment starting subsequently. For this purpose, stock solutions in DMSO or sterile H_2_O were serially diluted 1:1 in the respective medium, with the highest concentrations not containing more than 1% (v/v) organic solvent before application to the cells. 100 µL/well of these dilutions were added in triplicates for both, single and combined drugs. After a 72-h treatment, 20 µL of a 440 µM resazurin sodium stock (in PBS) were added to each well and N87, HCT116, as well as HT29 spheroids were stained for another 24 h period at 37 °C. MKN45 spheroids were stained for 6 h only, due to their fast metabolization of resazurin. For all four cell lines, measurements were conducted after a 96-h treatment. After the staining process, fluorescence values (ex/em = 530/620 nm) were measured with a microplate reader (SynergyHT, *BioTek*). IC_50_ values were interpolated with *Gen5 3.10* software from concentration-effect curves based on nine concentrations each from at least three independent experiments.

For combinations of established chemotherapeutics with BOLD-100, equimolar and IC_50_-dependent molarity ratios were tested under the same conditions. Only simultaneously combined drug treatments were tested, but not consecutive treatments, due to high risk of spheroid loss by exchanging the medium.

### Evaluation of combination effects

For all drug combinations tested, synergistic, additive or antagonistic effects were evaluated in order to give insight into whether drugs enhance or counteract each other’s cytotoxic impact. For this purpose, combination indices (CIs) were calculated from concentration-effect curves by using *Compu-Syn* software with algorithms established by Chou and Talalay [27]. Therefore, obtained effect values (= f_a_ (100 - % viability)/100)) at their respective concentrations were entered for all single drug as well as all combined drug treatments. Due to their different primary modes of action, we assumed mutually non-exclusive effects between BOLD-100 and all other chemotherapeutics. With these more cautious settings, the software calculated CI values for the respective effect level (f_a_).

### Morphology and growth properties of treated spheroids

For monitoring drug impact on growth and morphology of spheroids (as well as for the experiments described in the sections below), 0.25 × 10^3^ and 2.5 × 10^3^ cells in 100 µL per well were seeded for the gastric MKN45 and N87 cell lines, respectively, and 0.5 × 10^3^ cells in 100 µL per well for the colorectal cell lines HCT116 and HT29 into ULA plates. All spheroids were allowed to form for 96 h, with treatment starting subsequently. The four models were treated with the same concentrations of 20 µM and 200 µM of the respective single drug or combination with BOLD-100 (1:1 molar ratio) for 96 h. Organic solvent concentrations did not exceed 0.5% (v/v) when applied to the cells. Images of spheroids were taken with an Olympus (*CKX41)* inverse microscope with 4× objective magnification, and a ColorView camera before and after treatment. Diameters of formed aggregates and spheroids were measured in two orthogonal directions with the *CellF* software for statistical evaluation with at least three replicates per condition.

### Effects on reactive oxygen species levels

Six spheroids per condition were pooled in 1.5 mL Eppendorf tubes and centrifuged at 300 g for 3 min. The supernatant was removed, and spheroids were washed 1× with 500 µL PBS. After repeated centrifugation at 300 g for 3 min, the washing solution was discarded and 100 µL dichlorofluorescin-diacetate (DCFH-DA, *Sigma Aldrich*, 2.5 mM stock in DMSO diluted 1:100 in PBS) were added to the spheroids for 1 h at 37 °C. During staining, each well of a 96-well plate (flat bottom, *CytoOne*) was prepared with 100 µL of either single drugs or combinations in 40 µM and 400 µM concentrations. For negative controls, 100 µL of supplemented phenol-red-free Opti MEM (*Gibco*, + 1% FCS) were added. Tertbutylhydroperoxide (TBHP) was used as positive control, which was added as aqueous aliquot of 100 µL (4 mM).

Spheroids were washed with 500 µL PBS, centrifuged for 3 min and, upon addition of 100 µL supplemented phenol-red-free Opti MEM, transferred to the respective wells of the prepared 96-well plate. Thereby, concentrations were diluted to 20 µM and 200 µM (molar ratio for combinations was 1:1). The positive control was diluted to 2 mM. Fluorescence (ex/em = 485/516 nm) of spheroids was immediately measured in a plate reader (SynergyHT, *BioTek*) with *Gen5* software every 10 min for a total period of 2 h.

### Induction of apoptosis and necrosis

Spheroids were treated with 20 µM and 200 µM of the respective single drug or the combination with BOLD-100 (ratio 1:1) for 96 h. Afterwards, at least 6 spheroids per condition were pooled in 1.5 mL Eppendorf tubes and centrifuged at 300 g for 3 min. Spheroids were washed with 500 µL PBS and trypsinized with 200 µL (TrypLE Express, *Gibco*) per tube for 2–3 min at 37 °C. 600 µL of the respective supplemented medium were added to stop TrypLE Express activity. Single cells and cell aggregates were centrifuged and the supernatant was discarded. 250 µL FITC-conjugated annexin V, diluted 1:250 in binding buffer (10 mM HEPES/NaOH pH 7.4, 140 mM NaCl, 2.5 mM CaCl_2_ × 2H_2_O) were added per tube and incubated for 15 min at 37 °C. Meanwhile, 1 µL of propidium iodide solution (*Sigma Aldrich*, 2 mg/ml in sterile de-ionized H_2_O) per well was added to a round-bottom plate for flow cytometric analysis. After incubation, 200 µL cell suspension per condition were added to the plate and immediately analyzed with a flow cytometer (Guava, *Luminex*). Apoptotic and necrotic cells were evaluated with *FlowJo* software (v. 10.8.1) for at least three independent experiments.

## Results

### Formation of multicellular tumor spheroids

Two gastric (MKN45, N87) as well as two colorectal cancer (HCT116, HT29) cell lines were studied for their ability to form multicellular tumor spheroids in ultra-low-attachment plates. Varying cell numbers were tested to obtain cell aggregates or compact spheroids in sizes ranging between 250 μm and 800 μm on day 4, before drugs were applied, either alone or in combination (see the sections below).

**Fig. 1:**
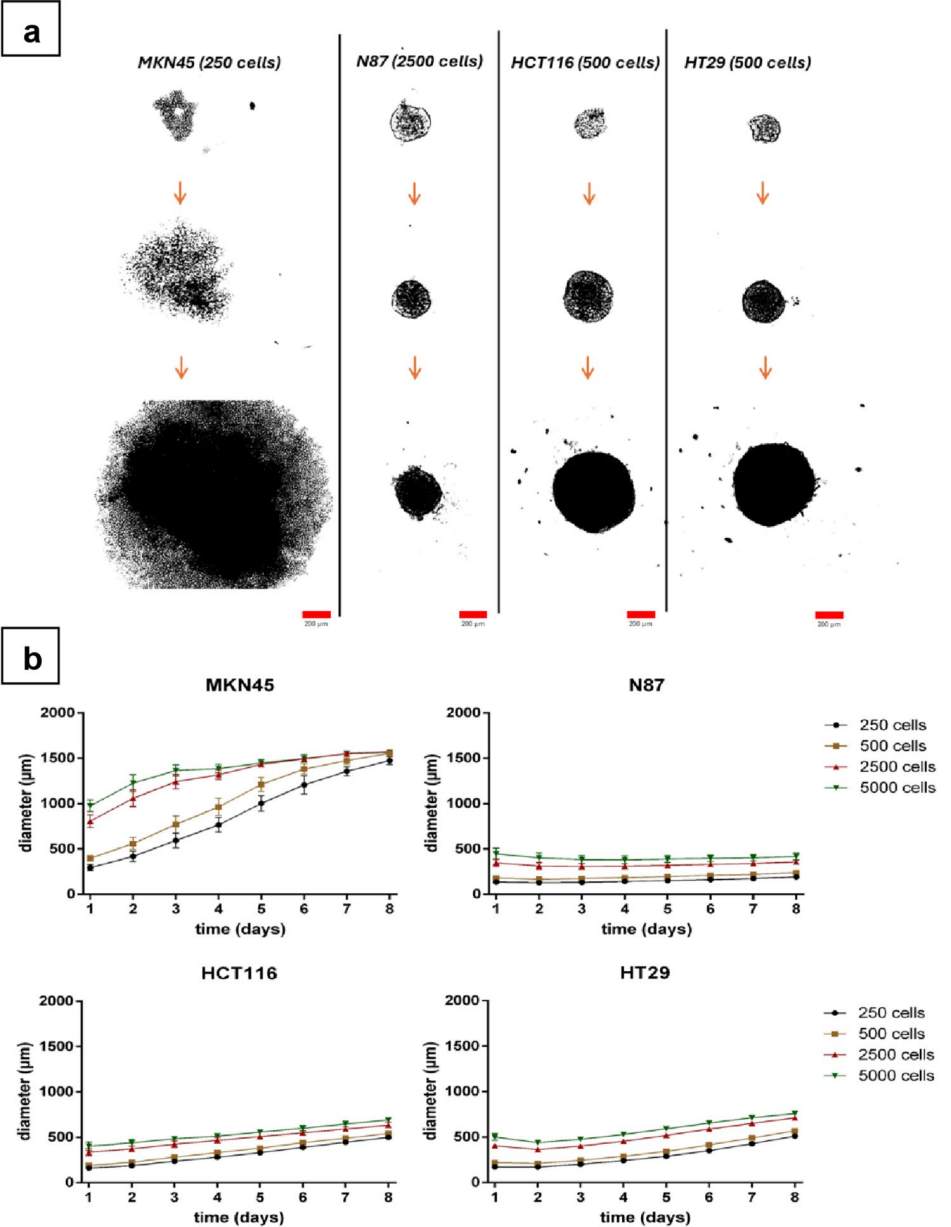
Growth behavior of multicellular tumor spheroids established from different gastrointestinal cell lines: Spheroids of varying cell numbers were grown in 96-well ultra-low attachment plates. Pictures of spheroids were taken every day for a total period of eight days with a ColorView camera attached to an inverse microscope (Olympus CKX41, 4 × objective magnification). (**a**) Representative images of gastric (MKN45, N87) and colorectal (HCT116, HT29) MCTSs were taken after one day (top), four days (middle) and eight days (bottom) of growth. Scale bars: 200 μm. (**b**) Diameters of spheroids were measured every day, for a total period of eight days for different initial cell numbers. (CellF software was used for analysis of three spheroids per cell number from at least three independent experiments.)

The gastric cell line MKN45 formed the biggest cell aggregates, independent on the cell number seeded, with clusters of cells more tightly bound in the core region and more loosely bound at the periphery. Spheroids were easy to dissociate by application of mechanical force. The lowest seeded cell number of 250 cells per well yielded aggregates of 766 ± 80 μm in diameter on day 4, which grew to sizes of 1476 ± 42 μm on day 8. Higher initial cell numbers led to cell aggregates bigger than 1500 μm in diameter, exceeding the field of view even at the lowest objective magnification (4 ×). Therefore, the growth curves seem to plateau at approximately 1500 μm for MKN45 spheroids (cf. Fig. 1 and S1). A completely different behavior was observed for N87 cells: Any seeded cell density resulted in spheroids either expanding only marginally or even remaining constant in size. This cell line formed compact spheroids until day 8; after compaction within the first 2–4 days, spheroids grew only by 30 μm to 80 μm in diameter till day 8 (Fig. 1 and S2). Due to their compactness, N87 spheroids were at least 3–8× smaller than those of MKN45, while MKN45 spheroids grew immensely due to their looser morphology.

Intermediate growth behavior could be found for the colorectal cancer cell lines. HCT116 MCTSs grew constantly during the period of 8 days, like MKN45 spheroids. A 1.8- to 1.4-fold size expansion was observed, reciprocally depending on initial cell density. For HT29 spheroids, compaction within the first two to three days was observed, especially for high cell numbers, as seen for N87 spheroids. Afterwards, they grew constantly and reached diameters between 510 ± 17 μm and 759 ± 3 μm on day 8. Both colorectal cell lines formed compact spheroids in the observation period (cf. Fig. 1 and Fig. S3, S4)*.*

### Characterization of spheroids by confocal microscopy

**Fig. 2:**
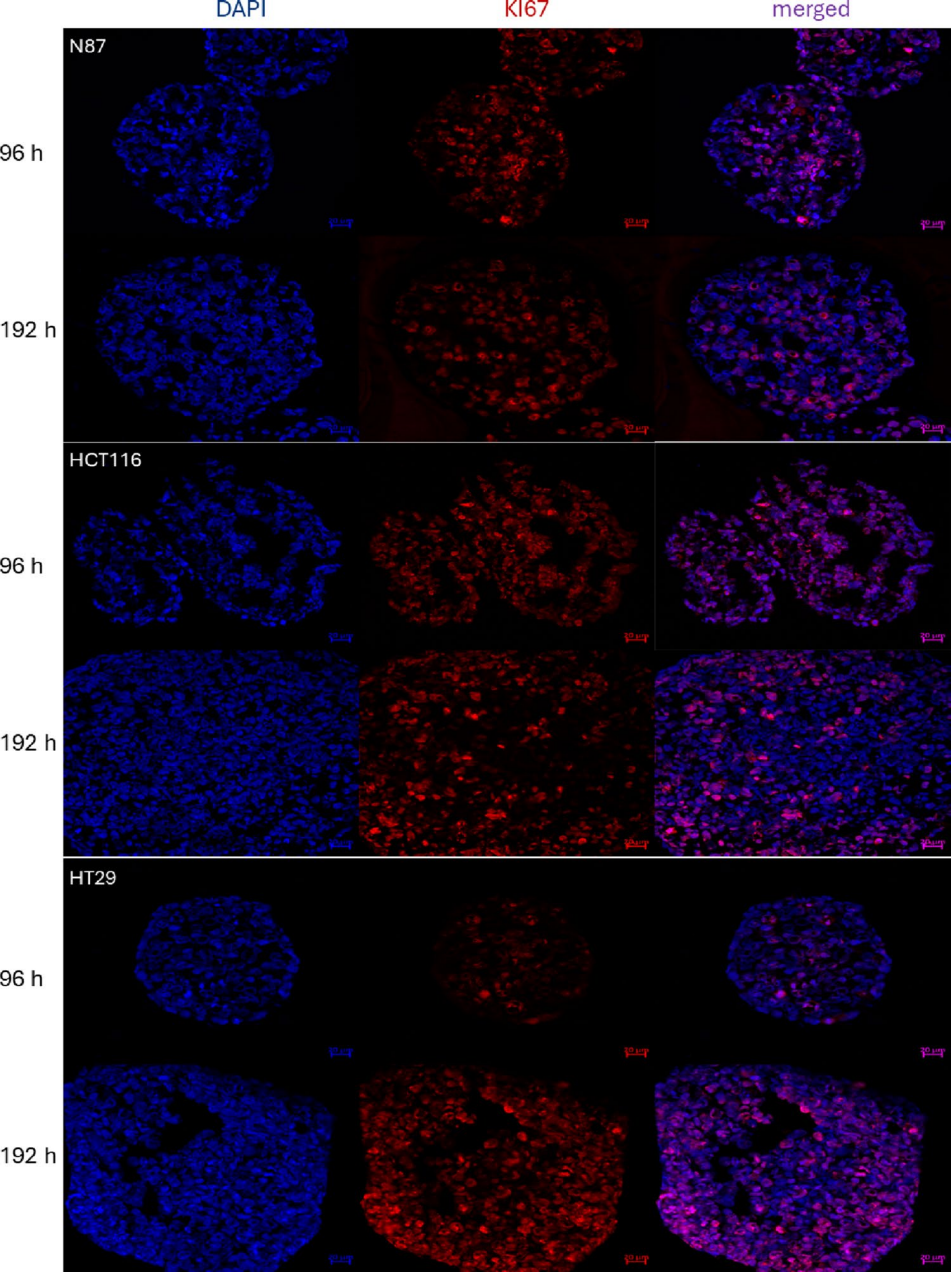
Representative, immunostained images of untreated spheroids taken by confocal microscopy: Samples were stained for nuclei (DAPI, blue) and proliferative cells (KI67, red) within the spheroids after 96 h and 192 h of growth with a Zeiss LSM800 confocal microscope and 20× objective magnification. Top panel: N87 spheroids; middle panel: HCT116 spheroids; bottom panel: HT29 spheroids. Scale bars: 20 μm

After growth and morphology features had been monitored, we were interested in attributes such as viability and hypoxia within the 3D models. For this reason, spheroids were embedded in TissueTek™ after 96 h as well as after 192 h, frozen to −20 °C and cut into 5 μm sections for staining with primary antibodies for proliferation (anti-KI67) or hypoxia (anti-HIF-1α). Prior to staining, samples were fixed, blocked and permeabilized. Figure 2 shows confocal microscopy images of N87, HCT116 and HT29 spheroid sections immunostained for the proliferation marker KI67 after 96 h and 192 h. All spheroid models revealed viable, proliferative cells throughout the entire sections independent of the time point under the chosen experimental conditions. Fig. S5 rather supports the absence of hypoxic conditions throughout untreated spheroids, as no pronounced staining for the hypoxia marker HIF-1α is discernible. Apparently, N87 spheroids are the only ones exhibiting signals of HIF-1α after eight days of growth, but a high background noise over the entire section questions their significance.

Sectioning MKN45 spheroids, seeded with 0.25 × 10^3^ cells per well, has been attempted after 96 h and 192 h as well. Due to their low compactness, though, preparation was unsuccessful at the steps of embedding and cutting.

### Cytotoxicity of single drugs as well as combined drugs in spheroid cell culture

We determined concentration-effect curves in multicellular tumor spheroids by means of the resazurin assay after a 96-h treatment. Table 1 lists the interpolated IC_50_ values for the single-drug and combined treatments of gastric (MKN45 and N87) and colorectal (HCT116 and HT29) tumor spheroids. The corresponding concentration-effect curves are depicted in Fig. S6-S9. Since many of the applied drugs were dissolved in DMSO, the impact of this solvent was checked after 96 h. In the gastric cancer models, DMSO had no effect up to 0.5% (v/v). In the colorectal cancer models, the effects of 0.5% (v/v) DMSO were minor (though not entirely neglectable), with percentages of viable cells declining to 90–93%, but it should be born in mind that this highest DMSO content is only relevant for the highest concentration of the respective drugs (see Fig. S17).

Regarding the single-drug treatments, the IC_50_ values varied in a wide range, even in spheroids derived from cells of comparable tissue origin. For instance, oxaliplatin was more than 3300-fold more active in MKN45 than in N87 spheroids, with respective IC_50_ values of 0.24 ± 0.05 µM and > 800 µM. A similar trend was observed for 5-FU and cisplatin. Cisplatin was tested in gastric cell lines only, due to its use in therapy of advanced gastric cancer [28]. Conversely, SN38, the active form of irinotecan, was applied to colorectal cancer spheroid models only. In HT29 spheroids, we tested concentrations of up to 800 µM of SN38, even though its hydrosolubility was limiting at the highest concentrations [22, 29]. We observed precipitation when suspension was not mixed continuously for concentrations higher than 20 µM, which should hence be considered with caution. To indicate its inactivity over the entire tested concentration range, we nevertheless stated “> 800 µM” for its IC_50_ in HT29 spheroids (cf. concentration-effect curve in ***Fig. S9***). Nevertheless, activity could be shown for HCT116 spheroids, which demonstrated the highest obtained activity of all tested drugs in MCTSs with an IC_50_ value of 2.5 ± 0.1 nM. BOLD-100, with a mode of action distinct from those of all other applied drugs, yielded IC_50_ values in moderate to high micromolar ranges. For both tissue origins, one cell line was markedly more sensitive (MKN45 and HCT116) to any treatment than the other one (N87 and HT29).

Dual drug combinations were first assessed in an equimolar manner, with the respective drugs being applied in the molar concentrations as BOLD-100. For MKN45 models, these approaches demonstrated that all tested combinations were either comparable to or less active than the single drugs. For example, 5-FU alone yielded an IC_50_ value of 2.2 µM; the combination with BOLD-100, though, resulted in an IC_50_ value of 6.4 µM, indicating lowered activity. For the cell lines N87 and HCT116, on the other hand, all tested equimolar combinations with BOLD-100 demonstrated higher activities than the single drugs. In HT29 spheroids, no noteworthy or determinable cytotoxicity was observed.

Apart from the equimolar setting, drug combinations were tested in roughly equipotent concentrations (= IC_50_-dependent ratios) as well. The latter revealed that the chemosensitive cell lines (MKN45 and HCT116) responded either comparably or even better than to single-drug treatments. The only exception was 5-FU in MKN45 spheroids, where single-drug activity was the same as that of the equipotent combination, whereas the equimolar combination with BOLD-100 showed lower cytotoxicity. For N87 spheroids, the 1:2 combination of BOLD-100 and oxaliplatin showed no improved effect compared to the equimolar combination. In HT29 spheroids, no noteworthy activities were observed for the combinations employing different molar ratios. For evaluating synergistic or antagonistic effects within the equimolar setting, combination indices (CIs) were calculated over a broad range in the next section.

**Table 1:** Cytotoxicity (IC_50_ in µM) of BOLD-100 and established chemotherapeutics in multicellular tumor spheroids of different cell lines: Single-drug cytotoxicity in gastric (MKN45, N87) and colorectal (HCT116, HT29) spheroids (top). Equimolarly combined drugs’ cytotoxicities in gastric and colorectal spheroids (middle). Combinations were tested in IC_50_-dependent ratios, derived from single-drug treatments in gastric and colorectal multicellular tumor spheroids (bottom). IC_50_ values were interpolated from concentration-effect curves obtained with the resazurin assay from at least three independent experiments. Means and SDs in µM; n. d. = not determinable; n. t. = not tested in the cell line

Single-drug treatment	MKN45	N87	HCT116	HT29
BOLD-100	151 ± 29	358 ± 37	152 ± 21	746 ± 55
Oxpt	0.24 ± 0.05	> 800	5.3 ± 3.9	n.d.
5-FU	2.2 ± 0.3	> 800	73 ± 70	n.d.
Cispt	2.3 ± 0.2	380 ± 20	n.t	n.t.
SN38	n.t.	n.t.	0.0025 ± 0.0001	> 800
		n.d. = not determinable		n.t. = not tested
Equimolar combinations	MKN45	N87	HCT116	HT29
B_Oxpt	0.28 ± 0.03	291 ± 14	3.6 ± 2.1	n.d.
B_5-FU	6.4 ± 2.1	372 ± 33	13.7 ± 3.6	n.d.
B_Cispt	2.5 ± 0.5	186 ± 27	n.t.	n.t.
B_SN38	n.t.	n.t.	0.0014 ± 0.0001	> 800
		n.d. = not determinable		n.t. = not tested
IC_50_-dependent combinations (molar ratios in brackets)	MKN45	N87	HCT116	HT29
B_Oxpt	0.23 ± 0.02 *(630:1)*	369 ± 31 *(1:2)*	1.6 ± 0.9 *(30:1)*	n.d. *(75:1)*
B_5-FU	2.2 ± 0.4 *(65:1)*	s.a. *(1:1)*	8.2 ± 2.4 *(2:1)*	n.d. *(7.5:1)*
B_Cispt	1.6 ± 0.3 *(65:1)*	s.a. *(1:1)*	n.t.	n.t.
B_SN38	n.t.	n.t.	0.0009 ± 0.0004 *(60*,*000:1)*	s.a. *(1:1)*
		n.d. = not determinable		n.t. = not tested

### Evaluation of combination effects

**Fig. 3:**
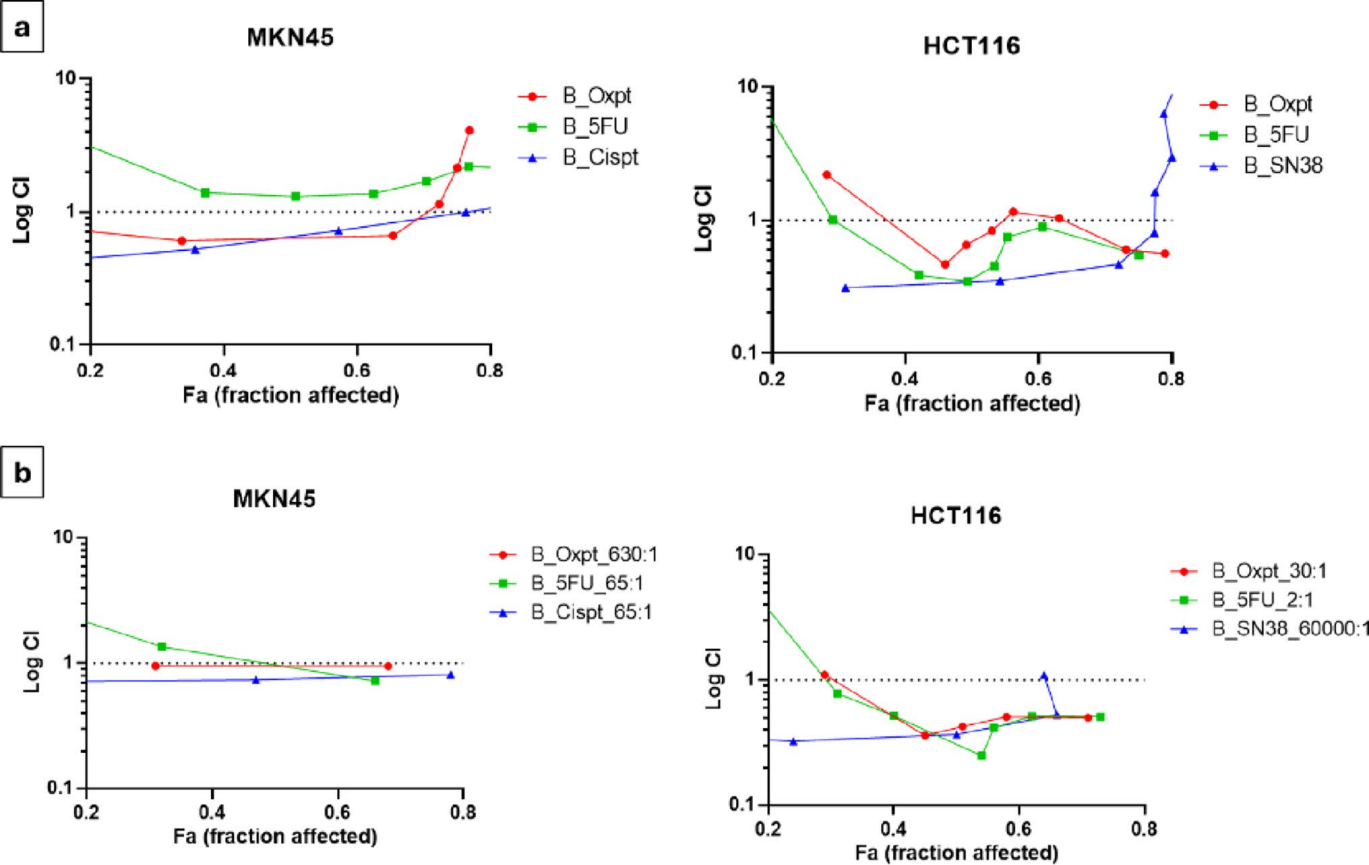
Combination indices (CIs) of drug combinations with BOLD-100 over the affected fraction-range between 0.2 and 0.8 for spheroids of the chemosensitive cell lines: Indices for equimolar combinations with BOLD-100 in gastric MKN45 and colorectal HCT116 multicellular tumor spheroids (**a**). Indices for IC_50_-dependent molar ratios in MKN45 and HCT116 MCTSs (**b**). Values < 1 indicate synergism, values ~ 1 indicate additivity, and values > 1 indicate antagonism; data were obtained from three independent experiments

Based on the values of obtained concentration-effect curves by means of the resazurin assay, combination indices (CIs) were calculated using *CompuSyn* software. For a better graphical overview in Fig. 3, those indices were depicted semi-logarithmically within a range of affected fractions between 0.2 and 0.8 (corresponding to IC_20_ and IC_80_). CIs < 1 indicate synergism, ~ 1 additivity and > 1 antagonism. Since most tested agents were inactive in the chemoresistant cell lines N87 and HT29, CIs were only determinable for the chemosensitive models MKN45 and HCT116. Figure 3a demonstrates the CIs of all equimolar combinations with BOLD-100 tested. Both platinum combinations with BOLD-100 showed slightly synergistic effects up to affected fractions of about 0.7; higher doses led to antagonistic effects in MKN45 spheroids. The combination of 5-FU with BOLD-100 caused minor antagonistic effects throughout the tested range. In HCT116 spheroids, all combinations yielded more or less synergistic behavior in a range of affected fractions between 0.4 and 0.7. Lower or higher doses seemed to act antagonistically. In Fig. 3b, combinations in IC_50_-dependent molar ratios showed similar but even more pronounced effects. Only 5-FU combined with BOLD-100 in MKN45 spheroids displayed a shift to slight synergism at a molar ratio of 65:1, indicating an overall positive effect of BOLD-100 in higher concentrations relative to the antimetabolite 5-FU. Due to the fact that treatment with IC_50_-dependent molar ratios of drugs hardly altered the overall trends of synergism or antagonism in our combination studies, we decided to conduct further experiments solely with equimolar combinations.

### Morphology and growth properties of treated spheroids

**Fig. 4:**
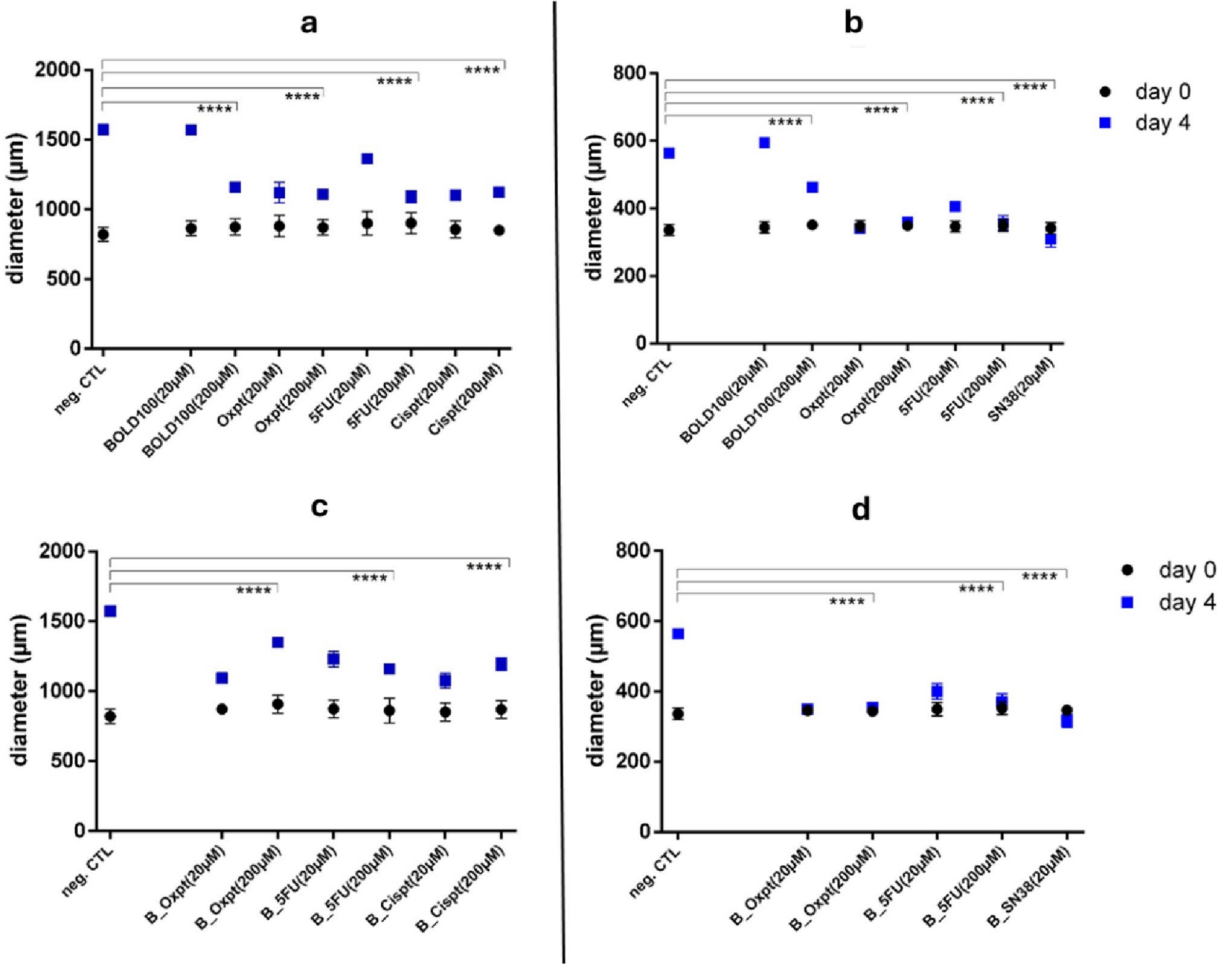
Spheroid growth properties upon single-drug and combined treatments (1:1) with chemotherapeutics plus BOLD-100 after 96 h: Inhibition of growth upon single-drug treatment with BOLD-100 in gastric MKN45 (**a**) and in colorectal HCT116 spheroids (**b**); growth inhibition upon combinational treatments in MKN45 **(c)** and in HCT116 multicellular tumor spheroids (**d**). Sizes were measured and analyzed with CellF software. All data were collected from three independent experiments; unpaired *t* test with Welch’s correction was conducted for statistical analysis (**** = *p*-value < 0.0001)

After 96 h of combinational treatments (ratio 1:1), a significant size reduction could be observed for at least one of the two tested concentrations for nearly all tested combinations performed in the four cell line models (Fig. 4). Usually, treated spheroids are expected to be either of the same size or smaller in diameter than untreated controls due to inhibitive effects on proliferation of any sort. We could observe this in many cases; however, our results also show that low concentrations frequently had a stronger impact on size than high concentrations of the same drug composition. Treatment with higher concentrations may even seem to enhance spheroid size, but we suppose that the integrity of compact 3D models got lost at higher drug concentrations, probably leading to loosening of cell cohesion on the outer rim. These effects were not as distinct in N87 and HCT116 spheroids, whereas MKN45 and HT29 seem to be affected more markedly (Fig. S10-S13). Basically, single-drug treatments exerted less pronounced effects compared to the combinational approaches (cf. Fig. 4 and S14). Only HCT116 spheroids demonstrated the expected effects for all combined drug treatments (Fig. 4b and d). In many models of the other cell lines, gastric as well as colorectal, treatment seemed to inhibit spheroid growth as well, but with slightly different effects (cf. Fig. 4c).

###  Formation of reactive oxygen species in a short-term approach

**Fig. 5:**
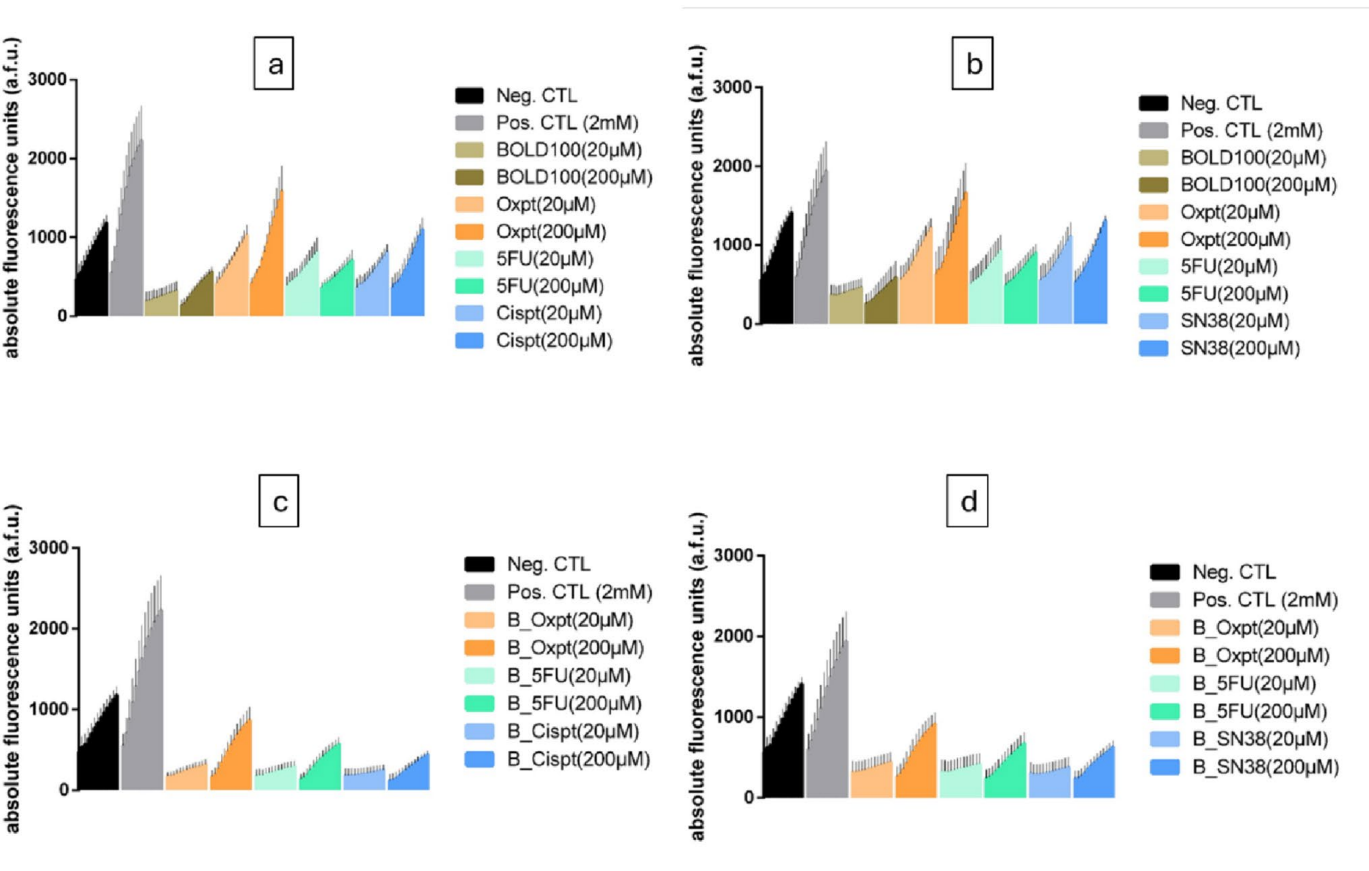
Reactive oxygen species (ROS) levels upon single-drug and equimolarly combined treatments with BOLD-100 in gastric and colorectal spheroid models measured via the DCFH-DA assay in a short-term approach: levels of ROS upon single-drug treatment measured in multicellular tumor spheroids formed by MKN45 (**a**) and HCT116 (**b**); effects on ROS upon combined drug application in MKN45 (**c**) and HCT116 spheroids (**d**). Data were obtained from three independent experiments, with six spheroids per condition tested. TBHP (tert-butylhydroperoxide) was used as positive control. Fluorescence was measured every 10 min for a total period of 2 h with a microplate reader

Reactive oxygen species (ROS) are important as cellular signaling molecules for proliferation, migration or angiogenesis. Imbalancing those levels may lead to DNA, protein or membrane damage and finally to cell death [30, 31]. Intracellular ROS might be affected by application of BOLD-100 due to its redox-chemical nature comprising a Ru(III) metal center, which can be reduced to the more active Ru(II). For this reason, we studied the effects of a short-term treatment in gastrointestinal multicellular tumor spheroids with the commonly used DCFH-DA assay [32]. As shown in Fig. 5c and d, ROS levels rose within a 2 h period of treatment and in a concentration-dependent manner for all tested equimolar combinations, even if levels shortly after the beginning of treatment were lower than those in the negative controls (see below). However, ROS proved hard to induce in MCTSs with the positive control tert-butylhydroperoxide (TBHP). Even with a concentration of 2 mM, levels were only raised up to 1.8- to 1.9-fold in the gastric cell lines (Fig. 5a and c, S15a and S15c, respectively), while a 1.4- to 1.5-fold increase was measurable for the colorectal models relative to the negative control (Fig. 5b and d, S15b and S15d).

Compared to negative controls, ROS levels seemed reduced at the onset of all treatments. This effect is strongest for (the colored) BOLD-100, but noticeable for the other single drugs as well and marginally concentration-dependent. As it remains unclear whether inner filter effects may account for these observations, we calculated both relative and absolute changes (the former potentially biased by any non-biological effect artificially lowering the baseline signal) for the evaluation of fluorescence data (Tables S1-S4). Relative increases of fluorescence suggest an induction of ROS for most single drugs (except 5-FU) and all equimolar combinations at their higher concentration in gastric cancer spheroids, as indicated by *F*_2h_/*F*_0h_ ratios higher than those in untreated controls. In colorectal cancer spheroids, the corresponding ratios suggest increases roughly comparable to untreated controls, with only oxaliplatin and oxaliplatin + BOLD-100 consistently exceeding them. When absolute changes in fluorescence are taken for data interpretation, oxaliplatin (at the higher concentration) appears as the only drug yielding higher ΔF values than untreated controls in all tested spheroid models, whereas BOLD-100 and 5-FU did not in any of these models. The latter also applies to all drug combinations, with oxaliplatin + BOLD-100 coming closest to the control values. Overall, oxaliplatin stands out as the most effective drug in terms of ROS induction, while its combination with BOLD-100 is the most likely to do so among the dual-drug treatments.

### Induction of apoptosis and necrosis in MCTSs upon treatment with single drugs and combinations with BOLD-100

**Fig. 6:**
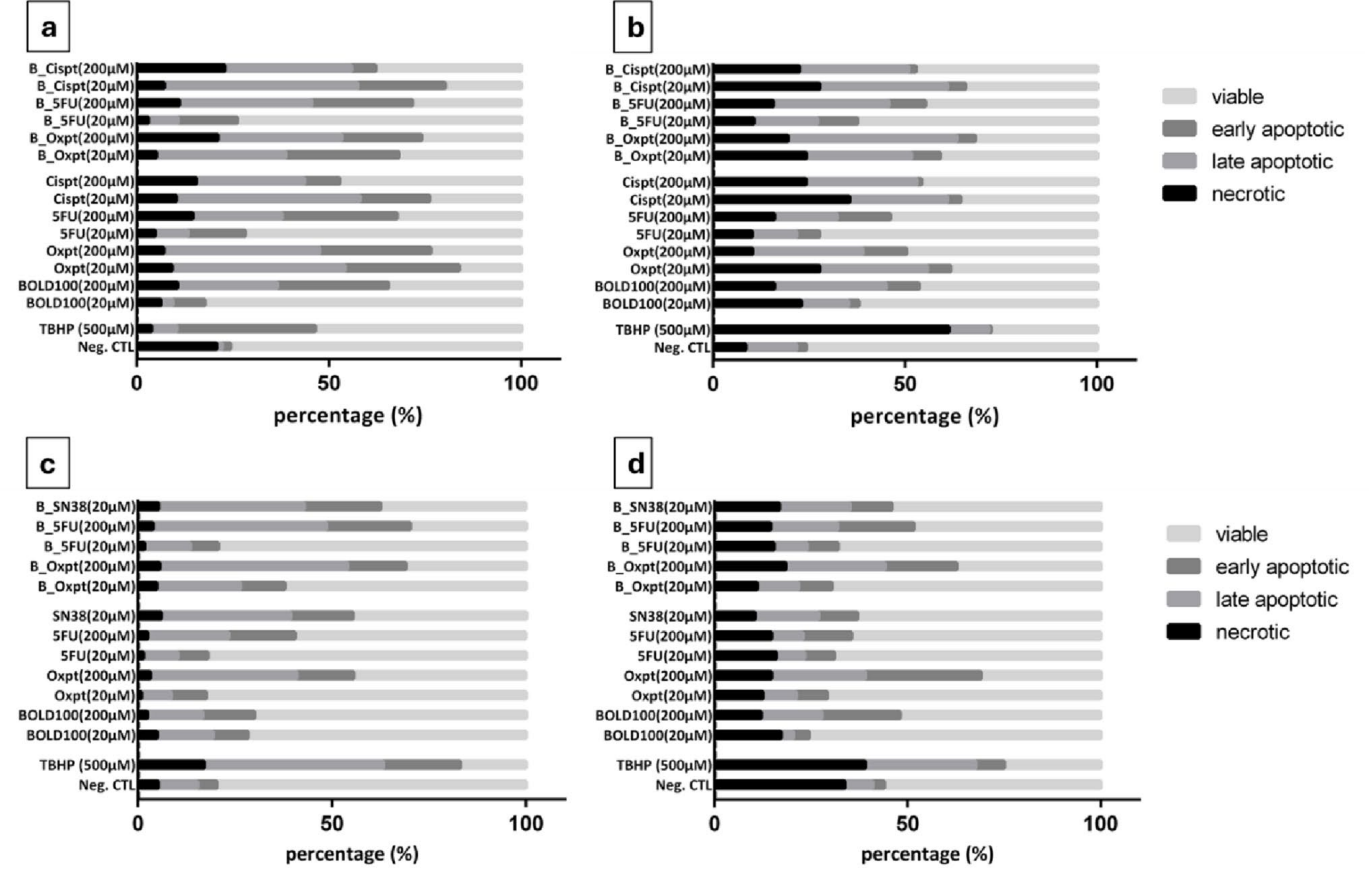
Apoptosis and necrosis induced upon single-drug treatment as well as in combination (1:1) with BOLD-100 in multicellular tumor spheroids after 96 h: MKN45 (**a**), N87 (**b**), HCT116 (**c**) and HT29 spheroids (**d**). All models were analyzed via flow cytometry by double-staining with annexin V/propidium iodide. As positive control, tert-butylhydroperoxide (TBHP) was used. Data were analyzed with FlowJo software based on five to six spheroids per condition in three independent experiments

Chemoresistance and apoptosis, a strictly organized form of cell death, are often reported to be pivotal cellular effects upon treatment with chemotherapeutics, either administered solely or in combinations. Genetic impacts, like p53- and GTSE1-regulation, or other cell types such as tumor associated macrophages, are often supposed to be involved in those processes [33–35]. In the annexin V/PI assay, we could show that apoptosis was indeed induced concentration-dependently in all the MCTS cultures (Fig. 6). For instance, up to 63% and 45% apoptotic cells were obtained by the combined treatment of BOLD-100 with oxaliplatin in HCT116 and HT29 spheroids, respectively. The antimetabolite 5-FU combined with BOLD-100 yielded 67% apoptotic cells for HCT116 and 37% for HT29 spheroids. BOLD-100 with SN38, applied only in a concentration of 20 µM, showed 58% and 29% apoptotic cells in HCT116 and HT29, indicating its high potency at least in HCT116 spheroids. For the gastric models MKN45 and N87, all chemotherapeutics in combination with BOLD-100 demonstrated also a differential induction of apoptosis, corresponding to their sensitivity in the cytotoxicity test. While MKN45 models yielded approximately 64% apoptotic cells for the combination with oxaliplatin, 60% with 5-FU and 73% with cisplatin, the same combinations only showed up to 49%, 40% and 38% apoptotic cells in MCTSs of N87. All obtained results are comparable between single-drug and combined drug testings, indicating that BOLD-100, applied as co-drug, alters the extent of apoptosis only marginally, even though its impact as single drug is usually remarkable, especially for MKN45 spheroids with up to 55% apoptotic cells induced (Fig. 6a).

Beside early and late apoptotic cells present in the four tested models, it is noteworthy that chemoresistant cell lines (N87 and HT29; Fig. 6b and d) seemed to overall yield more necrotic cells upon treatment than the chemosensitive cell lines (MKN45 and HCT116; Fig. 6a and c), independent of whether a single-drug or combined treatment was applied. Untreated N87 and HCT116 controls revealed lower percentages of apoptotic or necrotic cells than most of the treated samples, whereas MKN45 and HT29 controls showed even higher amounts of necrotic cells than the treated MCTs. For the latter two cell lines, this might be explained by the fact that controls were larger, contained more cells overall than treated spheroids after 96 h and were affected more distinctly in terms of their growth (cf. Fig. S10-S13).

## Discussion

Multicellular tumor spheroids have emerged as advanced models for anticancer drug screening. Their three-dimensional structure consisting of different layers mimics the in-vivo situation more closely than monolayer cultures, making them an excellent complement to conventional cell culture techniques. BOLD-100, a Ru-based pro-drug in clinical studies, was tested for the first time in combination with established chemotherapeutics in such spheroid models to investigate the impact on cytotoxic properties, the synergism or antagonism of combined treatment as well as the induction of ROS and apoptosis.

Assessing the antitumor effects of drug combinations in 3D models can be challenging and has for quite some time not been commonly pursued due to the special equipment required for spheroid formation. However, methodological progress towards high-throughput screening of drug combinations has been made recently [36]. Furthermore, there is a growing body of literature reporting the use of 3D cell culture models for the preclinical evaluation of substance combinations encompassing novel anticancer compounds and established drugs. For example, Yusoh et al. studied the effects of novel ruthenium(II) polypyridyl complexes in combination with the poly(ADB-ribose)polymerase (PARP) inhibitor olaparib on the growth of 3D triple-negative breast cancer models in agarose-coated microplates [37]. Lin et al. assessed the synergistic antiproliferative and ROS-enhancing effects of a Ru(II) polypyridyl complex in combination with doxorubicin in spheroids generated from breast cancer cells in commercially available microplates [38]. Similarly, we employed ready-to-use ultra-low attachment plates for the formation and treatment of gastrointestinal multicellular tumor spheroids.

Regarding the cytotoxic potency after 96 h of the tested single drugs and BOLD-100 in our models, the range of IC_50_ values varied enormously from low nanomolar to high micromolar levels, with SN38 being the most active compound in HCT116 spheroids. One of the two colorectal cancer models, namely HCT116, showed consistently higher chemosensitivity than the other, HT29. Since both models exhibit similar tight and compact morphology, reasons for the different sensitivity must be sought in other than these morphological features. Zhou et al. suggested reasons for those differences may be found in the different glycocalyx of these cell lines and especially in changes of intercellular glycoproteins in 3D models [39]. As for the gastric cancer cell lines, MKN45 spheroids were more sensitive to drug treatment than those of N87. Here, though, the two models varied in their spheroid morphology: While MKN45-derived MCTSs grew to unusually large sizes after 96 h pre-incubation even with small cell numbers seeded, spheroids formed by N87 cells remained quite small, no matter how many cells were seeded. For MKN45, most cells were only loosely attached to adjacent cells, resulting in a large-sized, spheroidal-shaped model. On the other hand, N87 cells formed robust, compact spheroids with smooth edges. Similar to the colorectal cancer models, glycoproteins on the cell membranes, relevant for cell-cell interactions, may be crucial for the different chemosensitivity of the gastric cancer models [40].

All tested models showed reduction in size after 96 h of incubation with drug combinations. However, no significant alterations were observed compared to single-drug treatments. Nevertheless, all drugs and their combinations with BOLD-100 induced morphological changes. Treated HCT116 and HT29 spheroids became smaller and less compact with more loosely attached cells at the outer rim than untreated controls, whereas treated MKN45 and N87 spheroids frequently showed irregular outlines. All equimolar drug combinations yielded pronounced synergism in HCT116 spheroids, similar to equipotent drug combinations, whereas only slight tendencies to synergism were observed in MKN45 spheroids, especially for the combination of 5-FU with BOLD-100.

Flocke et al. reported an impact of serum concentration on fluorescently measured ROS levels in 2D colorectal cancer cell cultures. A higher fluorescence was observed when BOLD-100 was applied with lower serum content in the medium [16]. Therefore, we used phenol-red-free medium with only 1% FCS (instead of 10%) for all 3D DCFH-DA approaches. The short-term combinational studies with BOLD-100 reported here showed no pronounced ROS induction at low drug concentrations, but partial increases of ROS levels at the higher drug concentrations, at least when relative (rather than absolute) changes within 2 h were considered. All cell lines showed decreased levels of ROS at the onset of treatment, compared to the respective negative controls. Higher drug concentrations raised those initial levels, which never exceeded those of untreated controls, though. This effect was also observable in all cancer cell lines tested, when BOLD-100 was applied as a single drug. The inherent color (brownish in DMSO as well as diluted in phenol-red-free medium) might also be a limiting factor when BOLD-100 solutions in concentrations higher than 100 µM are applied. While medium composition, serum content or even pH [16, 41, 42] may interfere with the outcome of fluorimetric methods, the inherent substance color may also result in quenching of the fluorescence signal. On the other hand, when BOLD-100 was tested in monolayer cultures (Fig. S16) of the same cell lines, a high concentration (200 µM) seemed necessary for ROS-inducing effects. In low concentrations, we also observed lower levels of ROS in all cell lines, which is in good accordance with a previous report [43]. Given that BOLD-100’s Ru(III) center can be activated by reduction to Ru(II), cells may activate BOLD-100 in low concentrations by consuming ROS, whereas a production of ROS may be observed at high concentrations.

To prove any oxidative impact with certainty, a long-term and more sensitive experimental setup might be desirable for 3D cultures, since the applied drugs have been frequently reported to induce ROS in monolayer cultures [44–48].

Our investigations on cell death by means of the annexin V/PI assay showed that both apoptotic and necrotic cells were present in all 3D models treated with single or combined drugs. Advantageously, though, combined drug treatments with BOLD-100 showed no enhanced necrosis induction in any of the cell lines. Basically, apoptotic cells were predominantly induced in the chemosensitive cell lines MKN45 and HCT116, while necrotic cells were scarcely detected, unlike the chemoresistant cell lines N87 and HT29, which responded with both enhanced apoptotic and necrotic cells. The reason for this observation has not been elucidated yet, but we assume that loosened cell-cell contacts and facilitated drug penetration into the spheroid models might be an important factor [39, 40].

## Conclusion

Anticancer drug screenings are mainly performed by means of monolayer-based cell culture approaches. Our study, though, focused on three-dimensional multicellular tumor spheroid models for the assessment of effects on essential cellular processes induced by the ruthenium-based pro-drug BOLD-100 in combination with clinically used cytostatic drugs in gastrointestinal tumor cell lines. The results showed that BOLD-100 is capable of synergizing with established chemotherapeutics in both gastric and colon cancer models, reconfirming the approach that had been chosen for clinical evaluation.

## Electronic Supplementary Material

Below is the link to the electronic supplementary material.

Supplementary Material 1

## Data Availability

The data, as far as not already available within the paper and its Supplementary Information, are available upon request.
