# Supplementary material for: Combinational studies of BOLD-100/KP1339 with established chemotherapeutics in gastrointestinal multicellular tumor spheroids
Source: Cancer Chemother Pharmacol. 2026 May 22;96(1):57. doi: 10.1007/s00280-026-04902-z (PMC13197383; doi:10.1007/s00280-026-04902-z)
Supplement: Supplementary file 1 — Supplementary Material 1 [file 280_2026_4902_MOESM1_ESM.pdf]

Combinational studies of BOLD-100/KP1339 with established chemotherapeutics on gastrointestinal multicellular tumor spheroids

Dominik Wenisch[a], Slavica Ždravac[a], Michael A. Jakupec[a,b], Franz Jirsa[c], Bernhard K. Keppler[a,b]

a Institute of Inorganic Chemistry, Faculty of Chemistry, University of Vienna, Währinger Strasse 42, 1090 Vienna, Austria

b Research Cluster "Translational Cancer Therapy Research", University of Vienna, Währinger Strasse 42, 1090 Vienna, Austria

c Institute of Inorganic Chemistry, Faculty of Chemistry, University of Vienna, Josef-Holaubek-Platz 2, 1090 Vienna, Austria

* Corresponding author: michael.jakupec@univie.ac.at, +43-1-4277-52610

Content

Formation of multicellular tumor spheroids....................2

Characterization of spheroids via confocal microscopy....................6

Cytotoxicity of single drugs as well as combined drugs in spheroid cell culture....................7

Changes in morphology and growth properties of treated spheroids....................11

Formation of reactive oxygen species in a short-term approach....................16

Reactive oxygen species in monolayer cultures....................17

Cytotoxicity of DMSO in spheroid cultures....................19

Formation of multicellular tumor spheroids

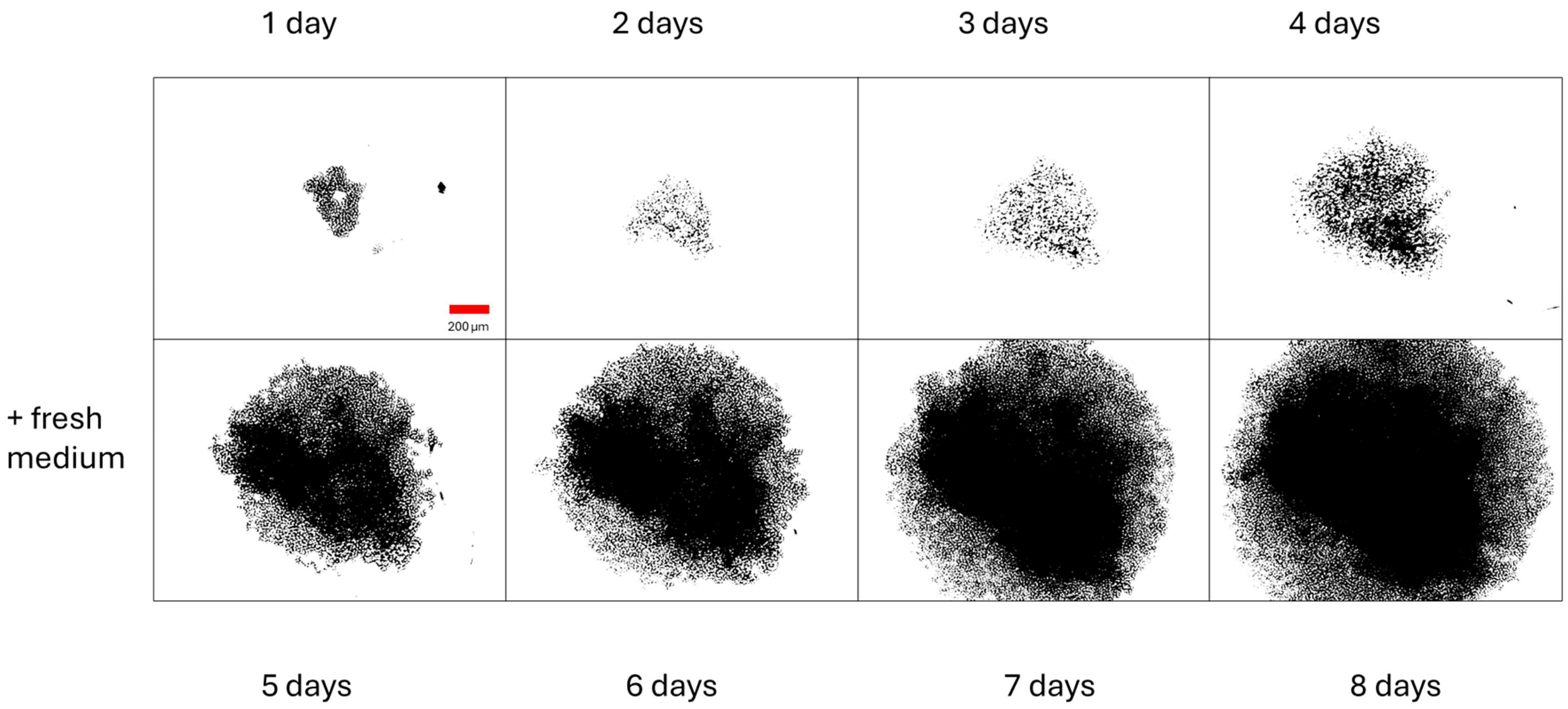

Figure S1: Representative images of multicellular tumor spheroids formed by gastric cancer cell line MKN45. Pictures were taken every day for a total period of 8 days with a ColorView camera attached to an inverse microscope (Olympus CKX41, 4× objective magnification). After 96 hours, the medium was renewed. Scale bars: 200 µm.

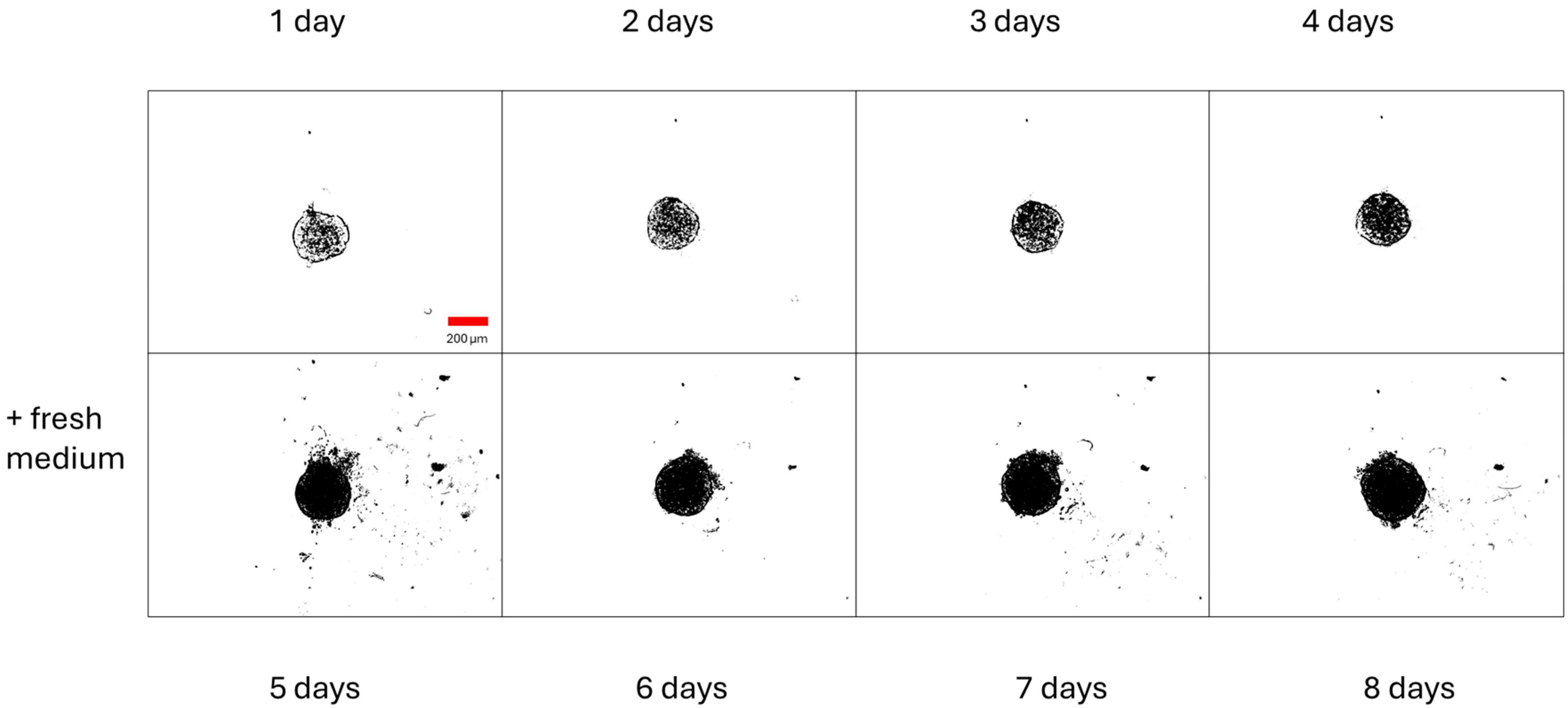

Figure S2: Representative images of multicellular tumor spheroids formed by gastric cancer cell line N87. Pictures were taken every day for a total period of 8 days with a ColorView camera attached to an inverse microscope (Olympus CKX41, 4× objective magnification). After 96 hours, the medium was renewed. Scale bars: 200 µm.

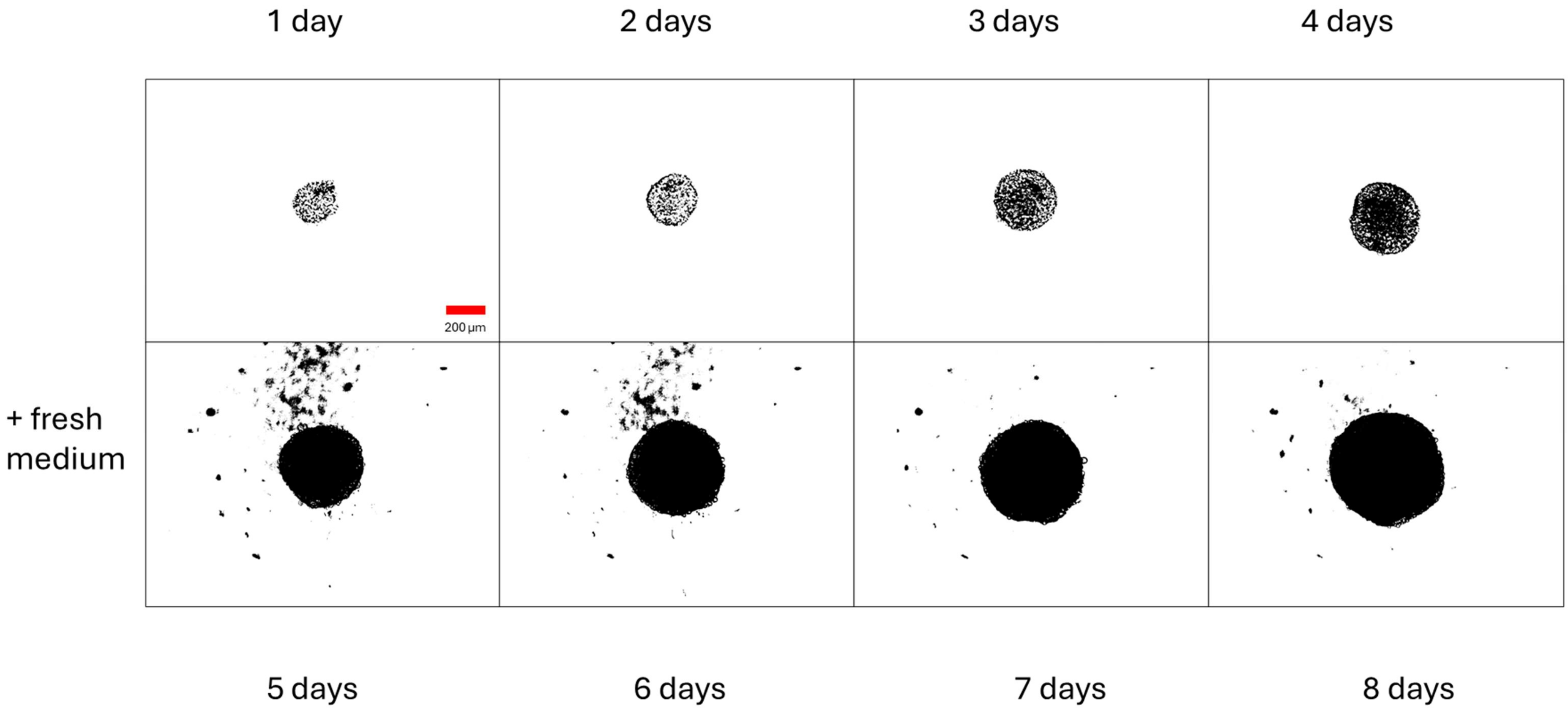

Figure S3: Representative images of multicellular tumor spheroids formed by colorectal cancer cell line HCT116. Pictures were taken every day for a total period of 8 days with a ColorView camera attached to an inverse microscope (Olympus CKX41, 4× objective magnification). After 96 hours, the medium was renewed. Scale bars: 200 µm.

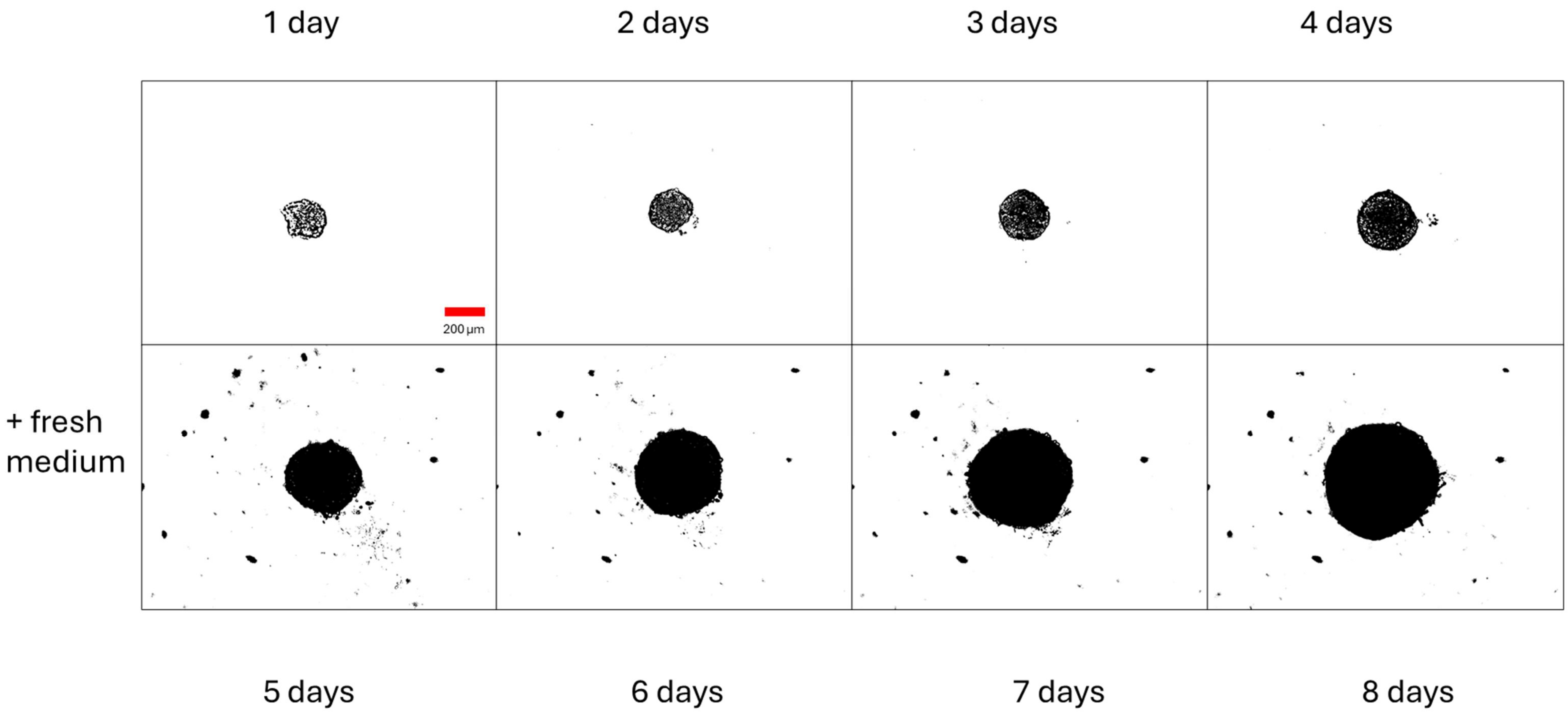

Figure S4: Representative images of multicellular tumor spheroids formed by colorectal cancer cell line HT29. Pictures were taken every day for a total period of 8 days with a ColorView camera attached to an inverse microscope (Olympus CKX41, 4× objective magnification). After 96 hours, the medium was renewed. Scale bars: 200 µm.

Characterization of spheroids via confocal microscopy

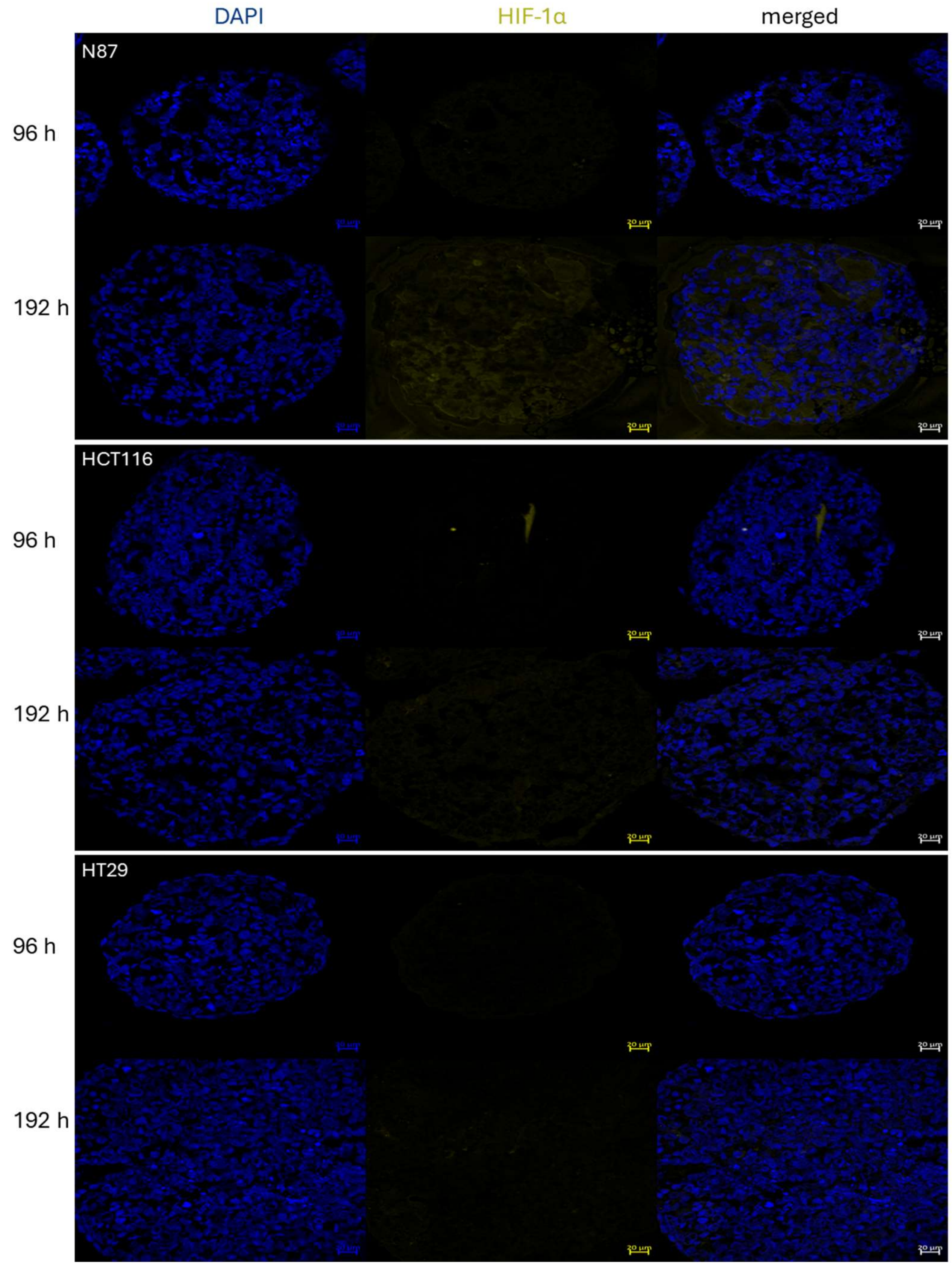

Figure S5: ***Representative, immunostained images of untreated spheroids taken via confocal microscopy.*** *Samples were stained for nuclei (DAPI, blue) and hypoxic cells (HIF-1α, yellow) within the spheroids after 96 h (4 days) and 192 h (8 days) of growth with a Zeiss LSM800 confocal microscope and 20× objective magnification. Top panel: N87 spheroids; middle panel: HCT116 models; bottom panel: HT29 spheroids. Scale bars: 20 µm.*

Cytotoxicity of single drugs as well as combined drugs in spheroid cell culture

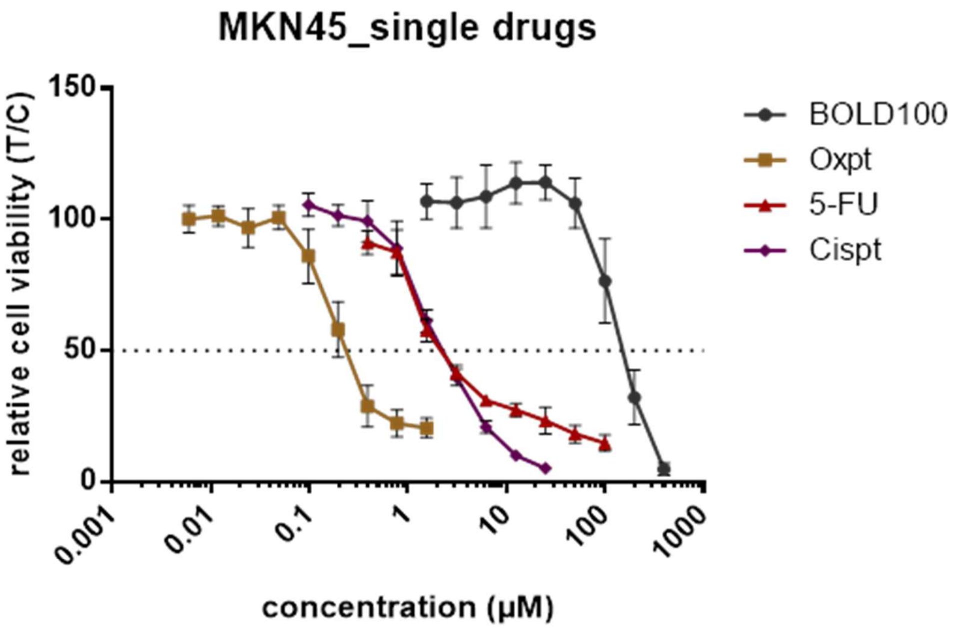

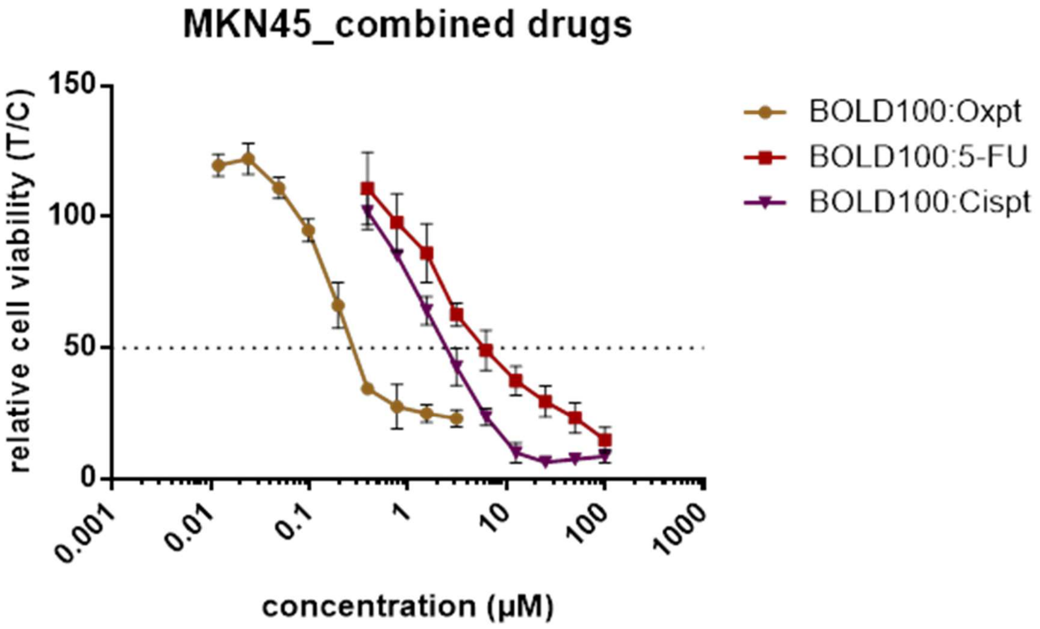

Figure S6: Concentration-effect curves of MKN45 spheroids evaluated with the resazurin assay after 96 hours. Single drugs and their combination (1:1) with BOLD-100 were tested and the half-maximal inhibtory concentration (IC_{50}) was extrapolated.

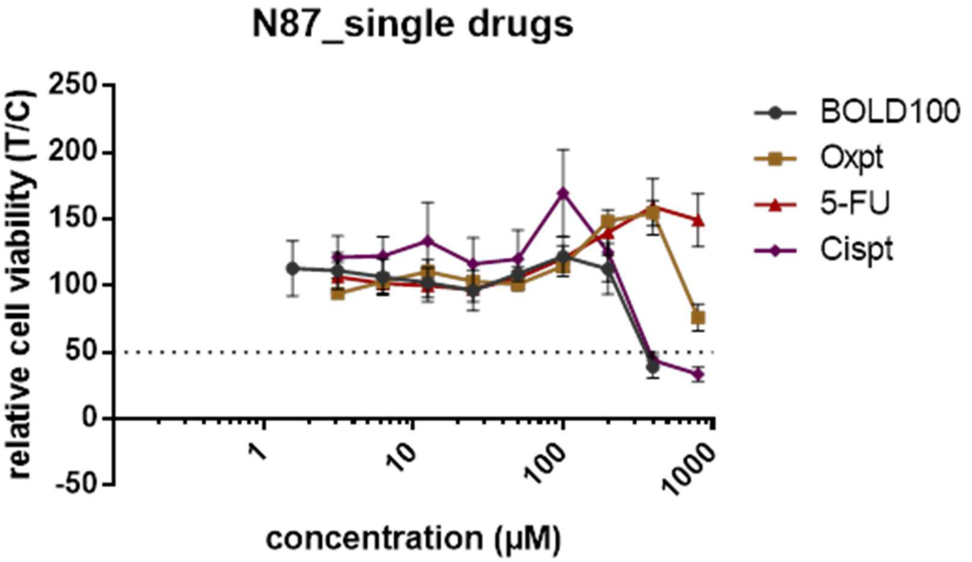

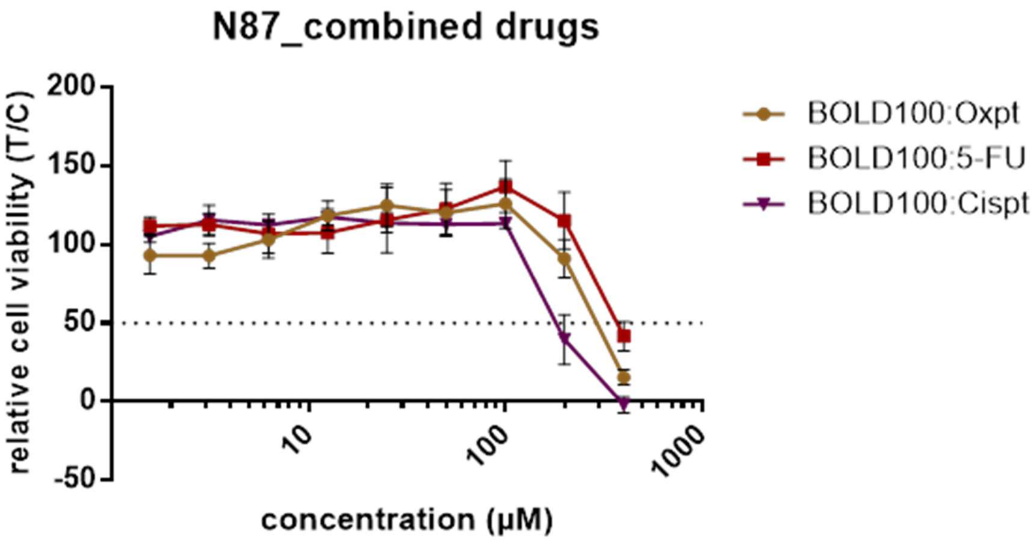

Figure S7: Concentration-effect curves of N87 spheroids evaluated with the resazurin assay after 96 hours. Single drugs and their combination (1:1) with BOLD-100 were tested and the half-maximal inhibtory concentration (IC_{50}) was extrapolated.

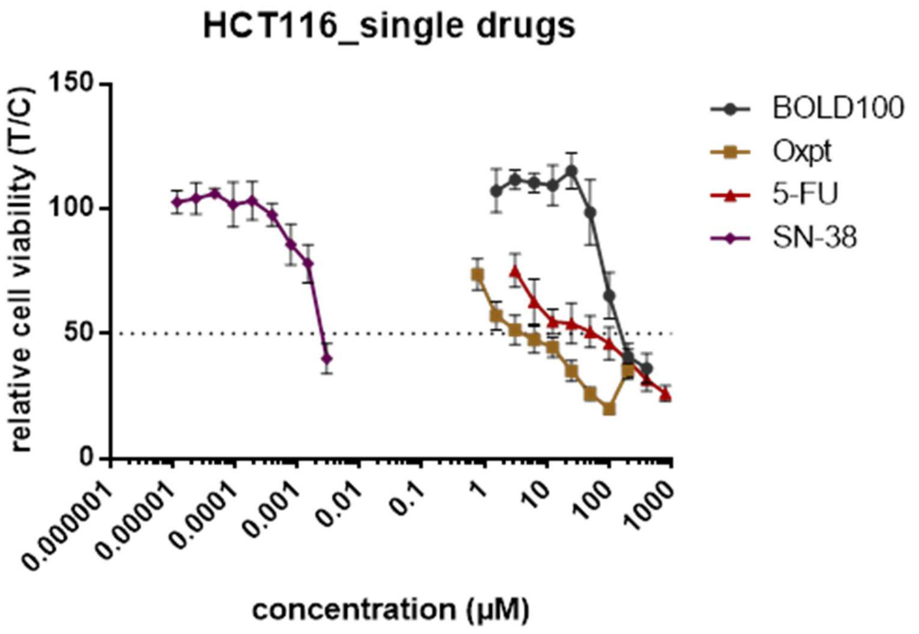

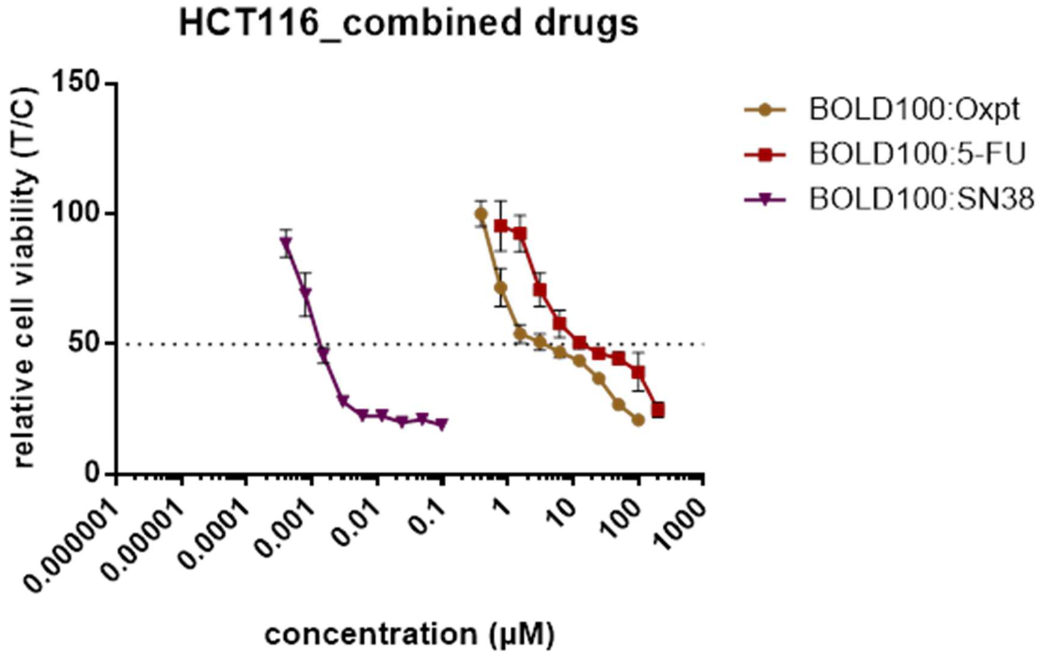

Figure S8: Concentration-effect curves of HCT116 spheroids evaluated with the resazurin assay after 96 hours. Single drugs and their combination (1:1) with BOLD-100 were tested and the half-maximal inhibtory concentration (IC_{50}) was extrapolated.

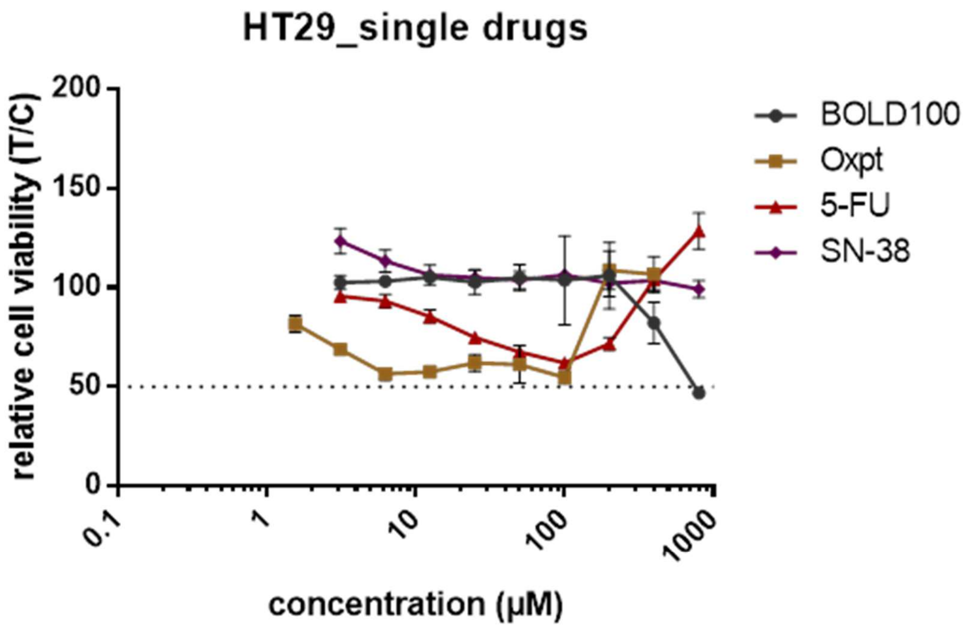

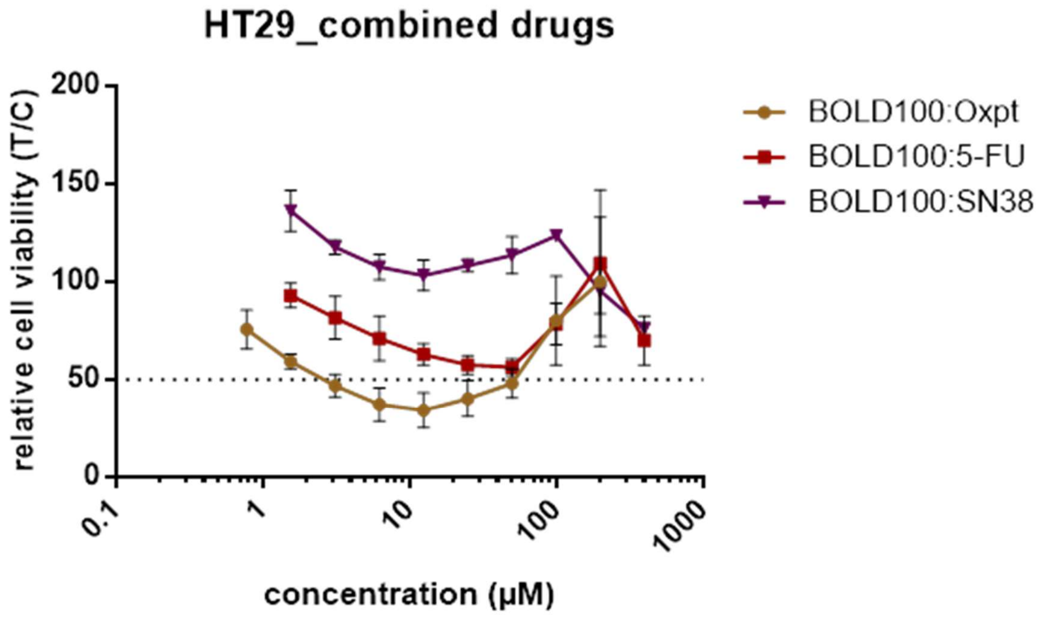

Figure S9: Concentration-effect curves of HT29 spheroids evaluated with the resazurin assay after 96 hours. Single drugs and their combination (1:1) with BOLD-100 were tested and the half-maximal inhibtory concentration (IC_{50}) was extrapolated.

Changes in morphology and growth properties of treated spheroids

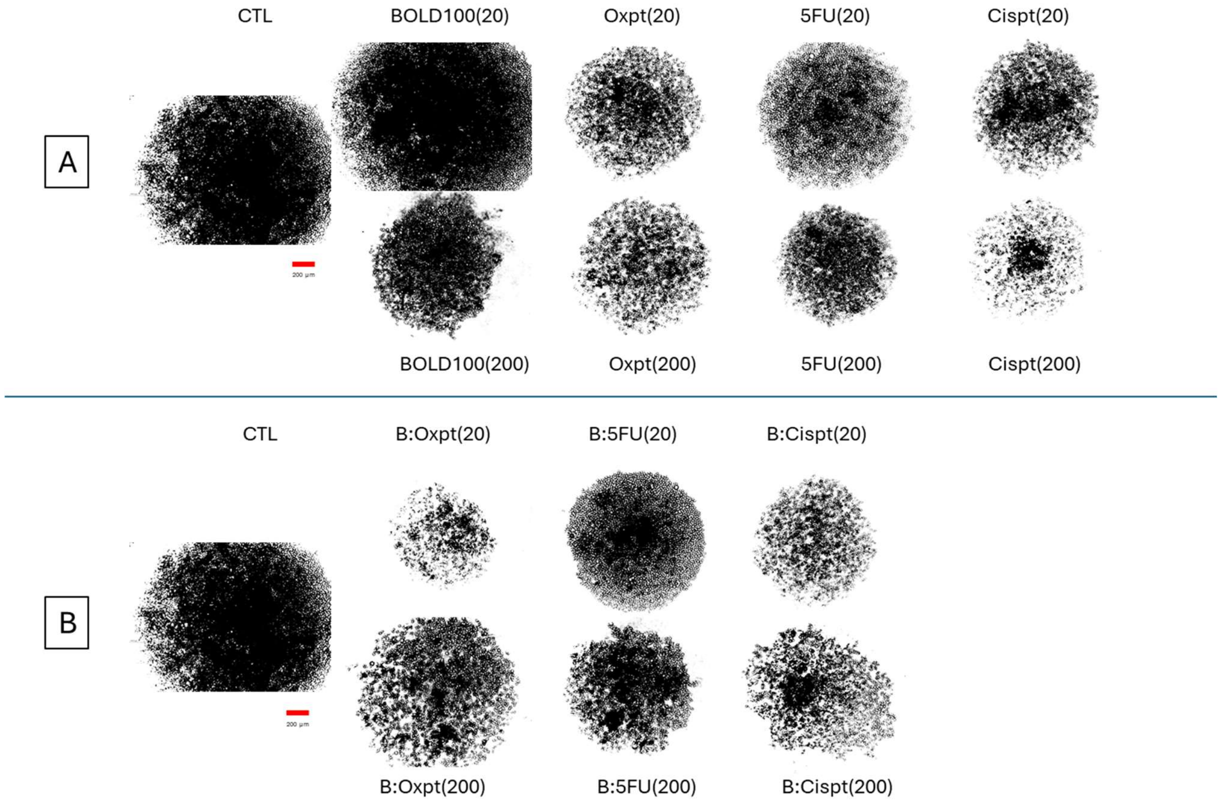

Figure S10: Representative pictures of treated gastric MKN45 spheroids after 96 hours. Single drug treatment of MCTSs **(A)**. Equimolarly-combined treatment with BOLD-100 **(B)**. Pictures were taken with a ColorView camera attached to an inverse microscope (Olympus CKX41, 4× objective magnification). Scale bars: 200 µm.

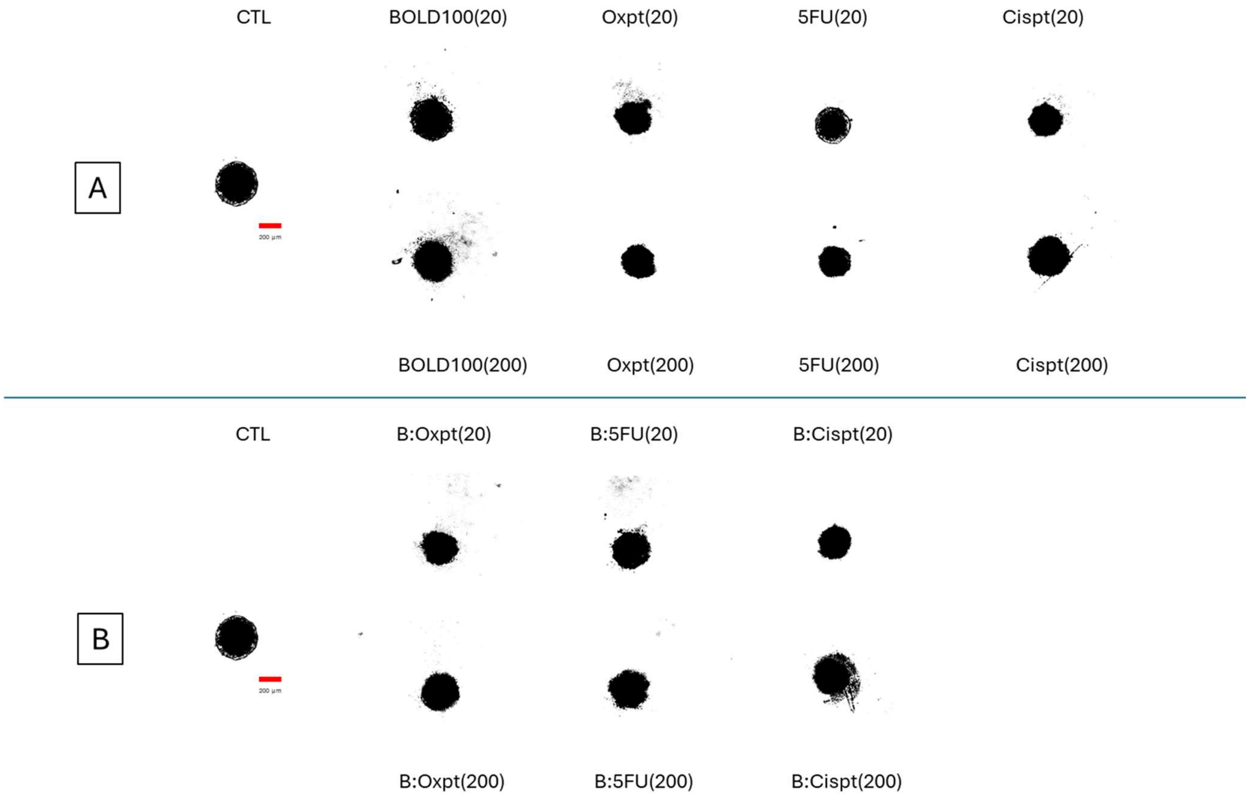

Figure S11: Representative pictures of treated gastric N87 spheroids after 96 hours. Single drug treatment of MCTSs **(A)**. Equimolarly-combined treatment with BOLD-100 **(B)**. Pictures were taken with a ColorView camera attached to an inverse microscope (Olympus CKX41, 4× objective magnification). Scale bars: 200 µm.

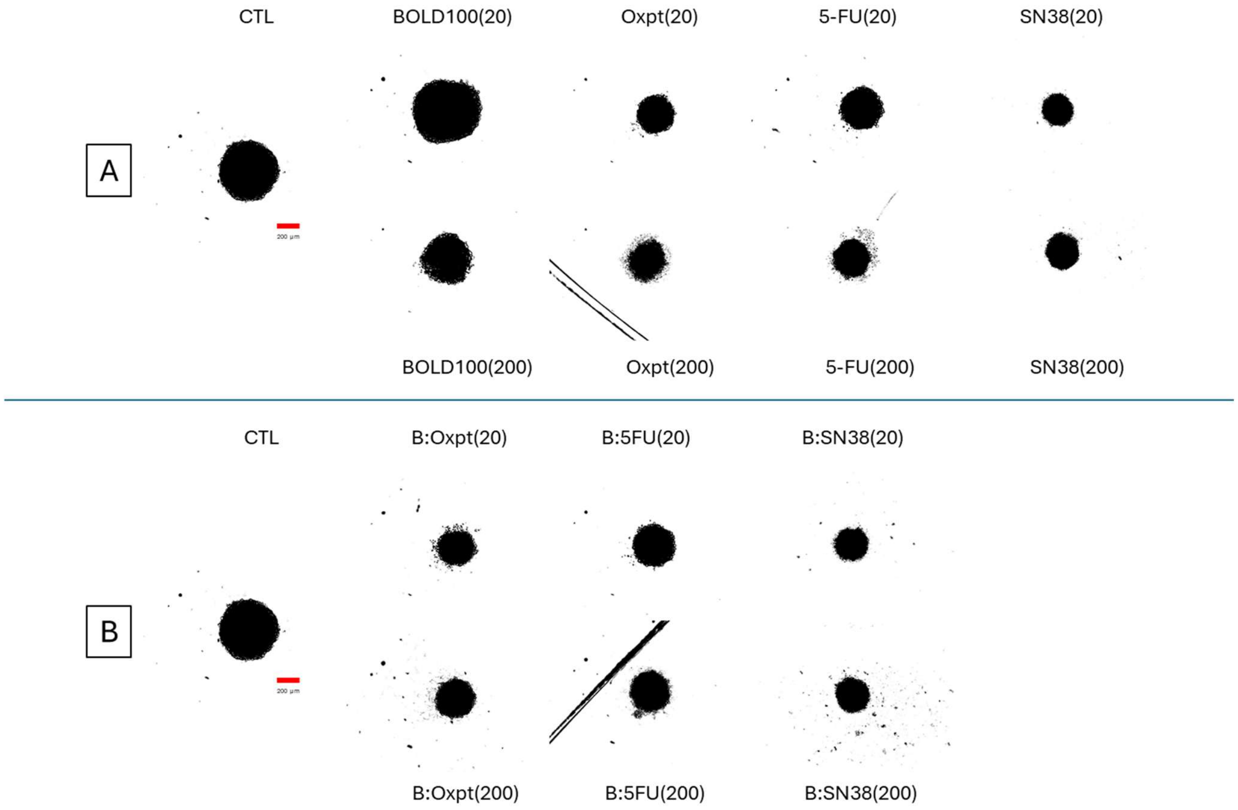

Figure S12: Representative pictures of treated colorectal HCT116 spheroids after 96 hours. Single drug treatment of MCTSs **(A)**. Equimolarly-combined treatment with BOLD-100 **(B)**. Pictures were taken with a ColorView camera attached to an inverse microscope (Olympus CKX41, 4× objective magnification). Scale bars: 200 µm.

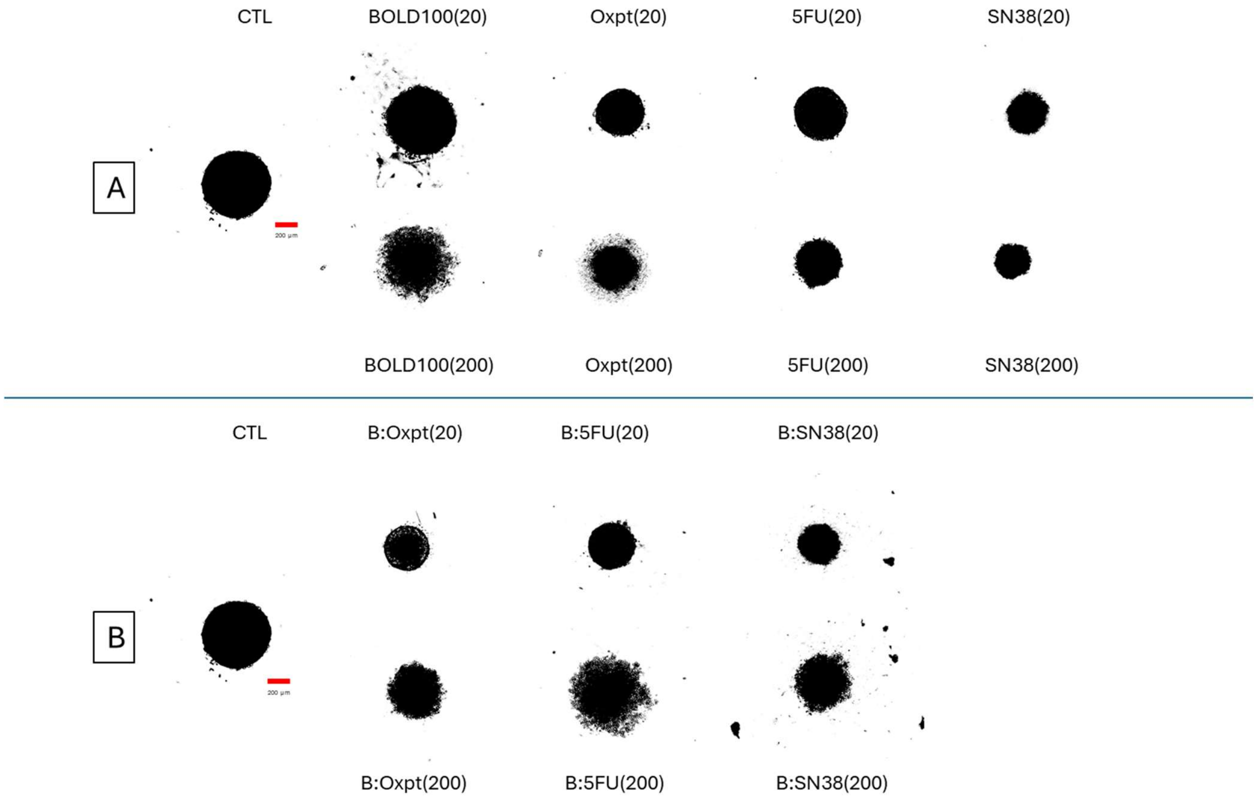

Figure S13: Representative pictures of treated colorectal HT29 spheroids after 96 hours. Single drug treatment of MCTSs **(A)**. Equimolarly-combined treatment with BOLD-100 **(B)**. Pictures were taken with a ColorView camera attached to an inverse microscope (Olympus CKX41, 4× objective magnification). Scale bars: 200 µm.

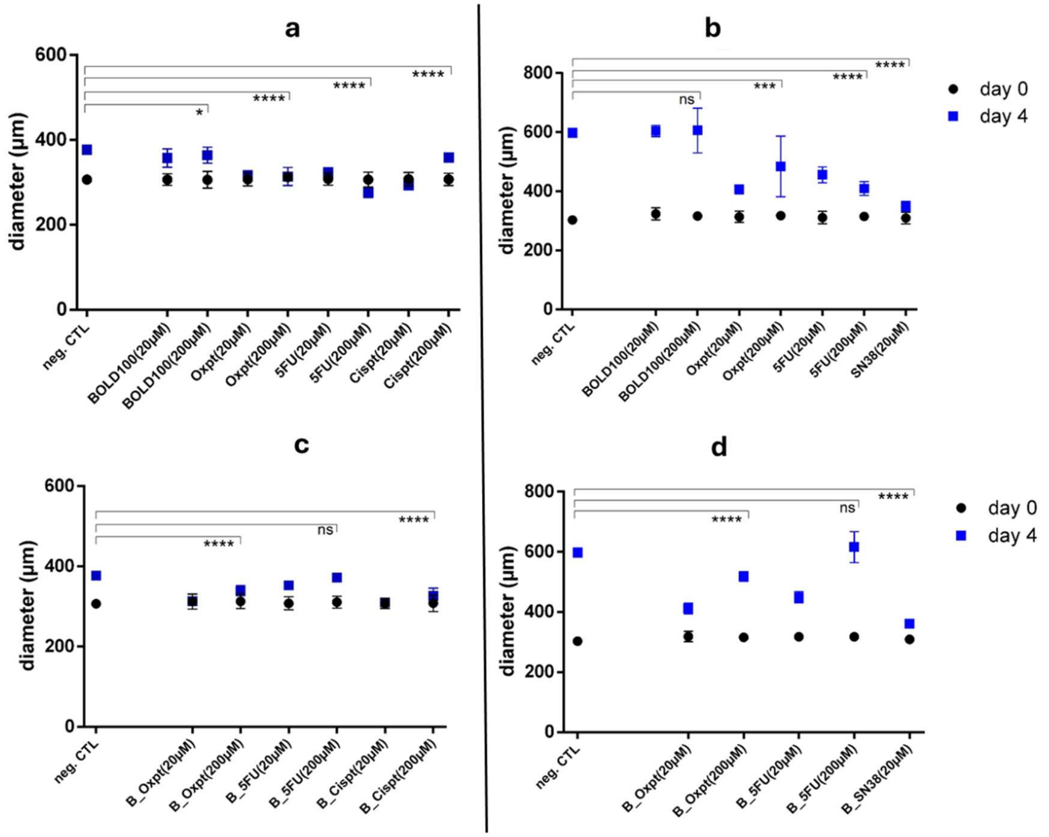

Figure S14: Spheroid growth properties upon single drug and combined treatment after 96 hours. *Inhibition of growth upon single drug treatment in gastric N87* ***(a)*** *and colorectal HT29 spheroids* ***(b)****; and effects upon combined drug treatments in N87* ***(c)*** *and in colorectal HT29 multicellular tumor spheroids* ***(d)****. Sizes were measured and analyzed with CellF software. All data were obtained from three independent experiments; unpaired t test with Welch's correction was conducted for statistical analysis. (**** = p-value < 0.0001; *** = p-value < 0.001; * = p-value < 0.1; ns = not significant)*

Formation of reactive oxygen species in a short-term approach

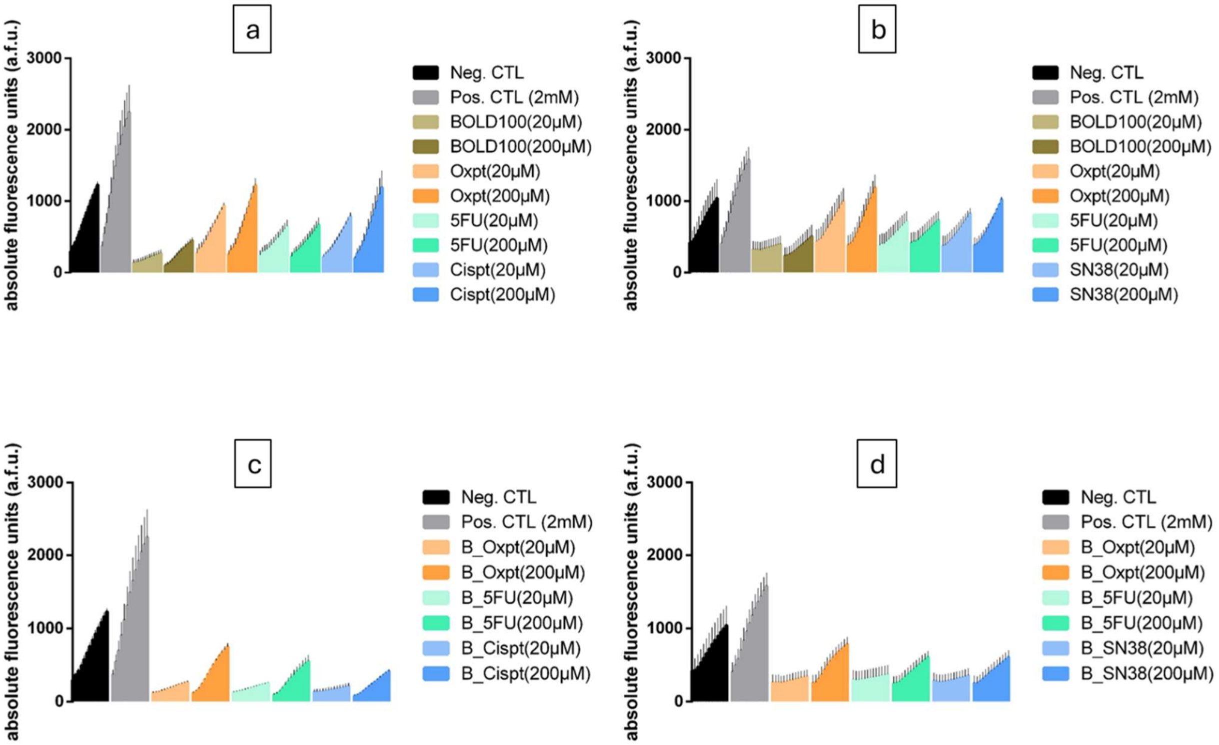

Figure S15: Formation of ROS in single-drug-treated and equimolarly combined drug-treated spheroids within 2 hours via DCFH-DA staining. ROS levels in single-drug-treated gastric N87 **(a)** and colorectal HT29 spheroids **(b)**; effects on ROS levels upon combined drug treatment in N87 **(c)** and HT29 spheroids **(d)**. Data were obtained from three independent experiments, with six spheroids per condition. TBHP (tert-butylhydroperoxide) was used as positive control. Fluorescence was measured every 10 min for a total period of 2 h on a microplate reader (SynergyHT, BioTek).

Table S1: Relative (F_{2h}/F_{0h}) and absolute changes of ROS-related fluorescence (ΔF) in MKN45 spheroids treated with two different concentrations. *Single drug treatment (a) and equimolarly combined treatment (b). Extracted from Fig. 5.*

**a)**	20 µM		200 µM	
	F_{2h}/F_{0h}	ΔF	F_{2h}/F_{0h}	ΔF
BOLD-100	1.7	130	4.0	427
Oxaliplatin	2.5	617	3.7	1168
5-FU	2.1	431	2.0	350
Cisplatin	2.2	451	3.0	736
Neg. CTL	2.5	713		
Pos. CTL	4.0	1677		

**b)**	20 µM		200 µM	
	F_{2h}/F_{0h}	ΔF		ΔF
B_Oxpt	1.8	147	5.2	702
B_5FU	1.7	119	4.2	435
B_Cispt	1.4	69	3.8	328
Neg. CTL	2.5	713		
Pos. CTL	4.0	1677		

Table S2: Relative (F_{2h}/F_{0h}) and absolute changes of ROS-related fluorescence (ΔF) in N87 spheroids treated with two different concentrations. *Single drug treatment (a) and equimolarly combined treatment (b). Extracted from Fig. S15.*

**a)**	20 µM		200 µM	
	F_{2h}/F_{0h}	ΔF	F_{2h}/F_{0h}	ΔF
BOLD-100	1.9	131	4.4	348
Oxaliplatin	3.4	669	4.8	969
5-FU	2.6	402	2.9	440
Cisplatin	3.6	570	5.8	988
Neg. CTL	4.2	934		
Pos. CTL	6.1	1876		

**b)**	20 µM		200 µM	
	F_{2h}/F_{0h}	ΔF	F_{2h}/F_{0h}	ΔF
B_Oxpt	2.3	150	6.3	634
B_5FU	2.0	128	5.8	458
B_Cispt	1.6	78	5.1	336
Neg. CTL	4.2	934		
Pos. CTL	6.1	1876		

Table S3: Relative (F_{2h}/F_{0h}) and absolute changes of ROS-related fluorescence (ΔF) in HCT116 spheroids treated with two different concentrations. *Single drug treatment (a) and equimolarly combined treatment (b). Extracted from Fig. 5.*

**a)**	20 µM		200 µM	
	F_{2h}/F_{0h}	ΔF	F_{2h}/F_{0h}	ΔF
BOLD-100	1.3	96	2.3	334
Oxaliplatin	2.2	661	2.6	1034
5-FU	1.8	424	1.8	414
SN38	2.0	556	2.5	782
Neg. CTL	2.5	848		
Pos. CTL	3.2	1339		

**b)**	20 µM		200 µM	
	F_{2h}/F_{0h}	ΔF	F_{2h}/F_{0h}	ΔF
B_Oxpt	1.4	123	3.4	651
B_5FU	1.3	93	2.8	435
B_SN38	1.2	73	2.6	393
Neg. CTL	2.5	848		
Pos. CTL	3.2	1339		

Table S4: Relative (F_{2h}/F_{0h}) and absolute changes of ROS-related fluorescence (ΔF) in HT29 spheroids treated with two different concentrations. *Single drug treatment (a) and equimolarly combined treatment (b). Extracted from Fig. S15.*

**a)**	20 µM		200 µM	
	F_{2h}/F_{0h}	ΔF	F_{2h}/F_{0h}	ΔF
BOLD-100	1.2	75	2.2	274
Oxaliplatin	2.3	566	3.1	804
5-FU	1.9	332	1.8	316
SN38	2.2	449	2.7	640
Neg. CTL	2.5	625		
Pos. CTL	3.9	1171		

**b)**	20 µM		200 µM	
	F_{2h}/F_{0h}	ΔF	F_{2h}/F_{0h}	ΔF
B_Oxpt	1.3	78	3.1	530
B_5FU	1.2	69	2.4	358
B_SN38	1.2	68	2.4	355
Neg. CTL	2.5	625		
Pos. CTL	3.9	1171		

Reactive oxygen species in monolayer cultures

Colon carcinoma cell lines HCT116 and HT29 were trypsinized for 3–5 min in a humidified incubator at 37 °C and under a 5% CO_2-atmosphere. Gastric cancer cell lines MKN45 and N87 were trypsinized for up to 8 min. McCoy's 5a medium (*Sigma-Aldrich*) supplemented with 10% heat-inactivated FCS (fetal calf serum; *Serana*) and 4 mM L-glutamin was added to stop trypsinization of colorectal cancer cells, while supplemented RPMI1640 medium was applied to the gastric cancer cells. Cell suspensions were centrifuged for 3 min at 1200 rpm. After aspiration of the supernatant, the cell pellet was resuspended in the respective supplemented medium. MKN45 and N87 cells were seeded in 100 µL aliquots in densities of 2.0 x 10^4 and 3.0 x 10^4 cells/well, respectively, whereas both HCT116 and HT29 cells were seeded in 100 µL aliquots of 2.5 x 10^4 cells/well, all into 96-well clear flat-bottom microplates. After incubation for 24 h, cells were washed with 200 µL Hanks' balanced salt solution (HBSS; *Sigma-Aldrich*; supplemented with 1% heat-inactivated FCS) and incubated with 100 µL/well of 25 µM 2',7'-dichlorofluorescin diacetate (DCFH-DA; in supplemented HBSS) for 45 min. After washing the cells with 200 µL of supplemented HBSS, BOLD-100 (from a 100% DMSO stock, serially diluted in supplemented phenol-red-free Opti-MEM (*Gibco*) supplemented with 1% heat-inactivated FCS) was added in 200 µl triplicates. Immediately after addition of the test compound, fluorescence (ex/em = 480/516 nm) was measured every 10 min for a total period of 2 h with a microplate reader (BioTek, Synergy HT). Blank-corrected values are represented relative to negative controls (treated with drug-free supplemented Opti-MEM) from at least three independent experiments.

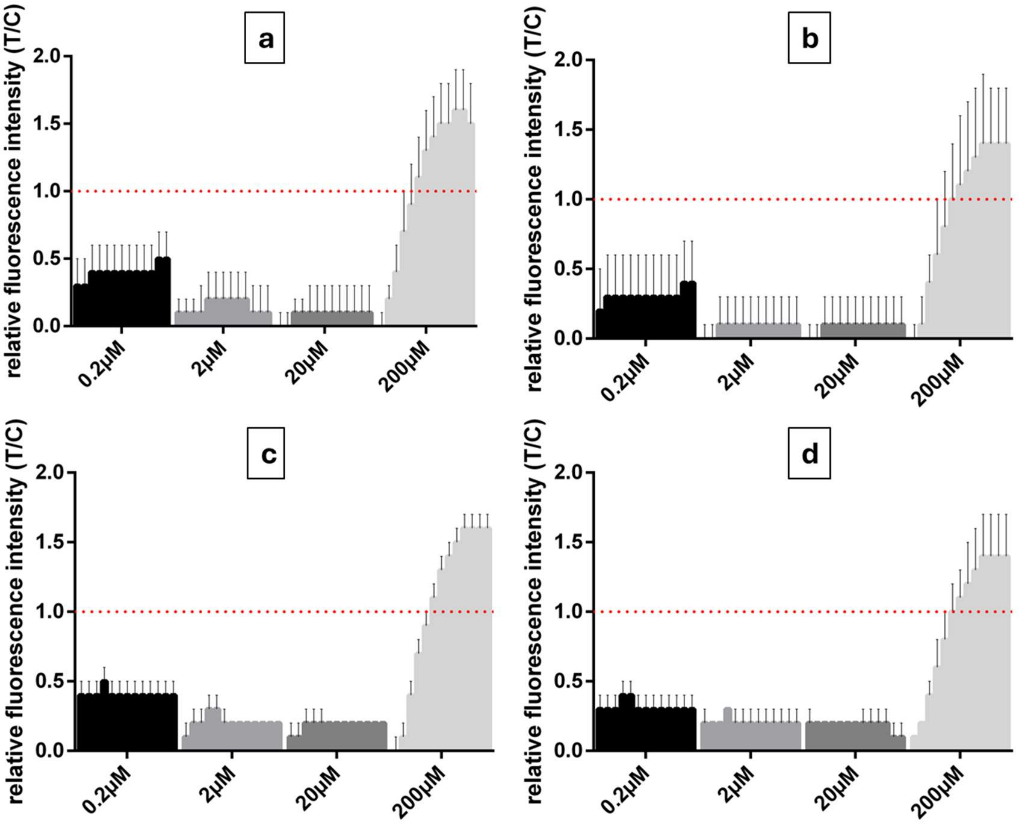

Figure S16: Generation of reactive oxygen species in monolayer cultures upon treatment with BOLD-100. Levels of ROS were measured by means of the DCFH-DA assay for monolayer cell cultures of MKN45 **(a)**, N87 **(b)**, HCT116 **(c)** and HT29 **(d)**. Data were obtained from three independent experiments. Fluorescence was measured every 10 min for a total period of 2 h on a microplate reader (SynergyHT, BioTek).

Cytotoxicity of DMSO in spheroid cultures

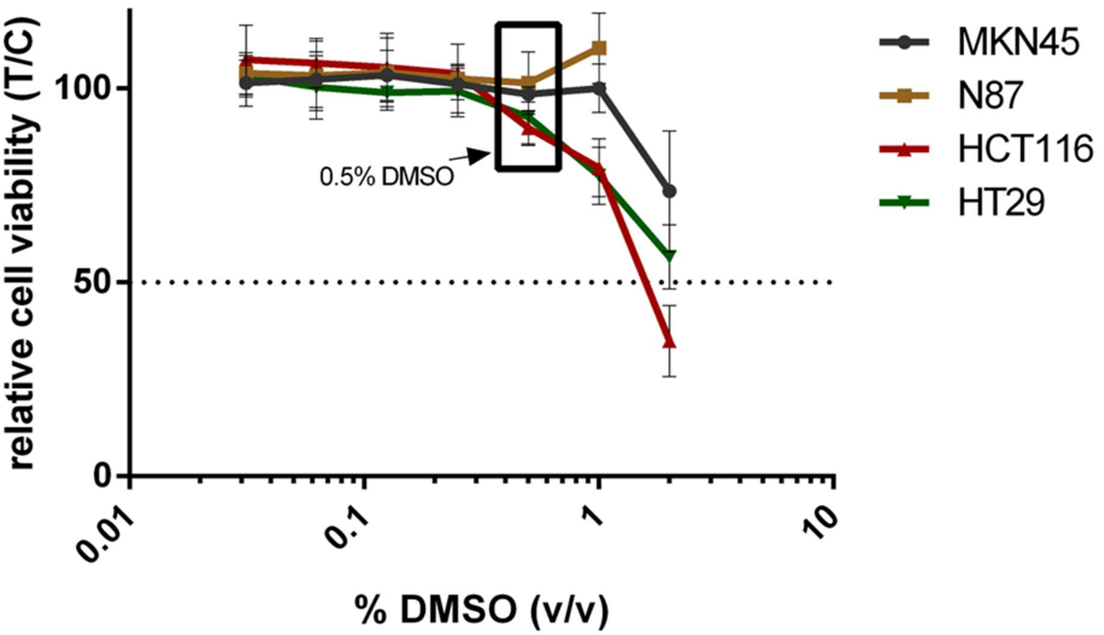

Figure S17: Cytotoxic effects of dimethylsulfoxide (DMSO) in 3D cell cultures after 96 h, determined by the resazurin assay. Data were obtained from at least three independent experiments for all cell lines. Cell numbers and culture conditions for the respective cell lines were the same as those mentioned in the main paper.
